# Chronic *Trichuris muris* Infection in C57BL/6 Mice Causes Significant Changes in Host Microbiota and Metabolome: Effects Reversed by Pathogen Clearance

**DOI:** 10.1371/journal.pone.0125945

**Published:** 2015-05-04

**Authors:** Ashley Houlden, Kelly S. Hayes, Allison J. Bancroft, John J. Worthington, Ping Wang, Richard K. Grencis, Ian S. Roberts

**Affiliations:** Faculty of Life Sciences, University of Manchester, Manchester, M13 9PT, United Kingdom; Purdue University, UNITED STATES

## Abstract

*Trichuris* species are a globally important and prevalent group of intestinal helminth parasites, in which *Trichuris muris* (mouse whipworm) is an ideal model for this disease. This paper describes the first ever highly controlled and comprehensive investigation into the effects of *T*. *muris* infection on the faecal microbiota of mice and the effects on the microbiota following successful clearance of the infection. Communities were profiled using DGGE, 454 pyrosequencing, and metabolomics. Changes in microbial composition occurred between 14 and 28 days post infection, resulting in significant changes in α and β- diversity. This impact was dominated by a reduction in the diversity and abundance of Bacteroidetes, specifically Prevotella and Parabacteroides. Metabolomic analysis of stool samples of infected mice at day 41 showed significant differences to uninfected controls with a significant increase in the levels of a number of essential amino acids and a reduction in breakdown of dietary plant derived carbohydrates. The significant reduction in weight gain by infected mice probably reflects these metabolic changes and the incomplete digestion of dietary polysaccharides. Following clearance of infection the intestinal microbiota underwent additional changes gradually transitioning by day 91 towards a microbiota of an uninfected animal. These data indicate that the changes in microbiota as a consequence of infection were transitory requiring the presence of the pathogen for maintenance. Interestingly this was not observed for all of the key immune cell populations associated with chronic *T*. *muris* infection. This reflects the highly regulated chronic response and potential lasting immunological consequences of dysbiosis in the microbiota. Thus infection of *T*. *muris* causes a significant and substantial impact on intestinal microbiota and digestive function of mice with affects in long term immune regulation.

## Introduction

Helminth infections have increasingly been recognised as a global health issue, predominantly affecting developing counties with in excess of 2 billion individuals infected worldwide [1]. *Trichuris* species are a highly prevalent and successful group of intestinal helminth parasites, with a diverse range of mammalian hosts [2]. The human whipworm, *Trichuris trichiura*, is estimated to infect over 600 million people worldwide causing chronic morbidity [1]. *Trichuris* species inhabit the caecum and colon of their hosts where they are in close association with the host microbiota [3] with strong interactions with the host immune system [4]. The relationship between commensal bacteria and their hosts has resulted from the co-evolution over millions of years. The mammalian intestine provides a stable temperature and access to nutrients thereby providing a suitable environment for bacterial growth, while at the same time the host microbiota facilitate the digestion of dietary macromolecules and provide signals essential for normal intestinal physiology [5,6]. The host needs to balance an ability to mount an effective immune response against intestinal pathogens while regulating the host’s immune responses to commensal bacteria [6]. Dysbiosis in this regulation has been linked to several intestinal diseases such as diabetes [7] Crohn's [8], inflammatory bowel disease [9], cancer [10], and even neurological diseases such as autism [11].

*Trichuris muris* (mouse whipworm) is an ideal model for studying *Trichuris* infections, pathology and associated immune responses as the life cycle is remarkably similar between species [12]. The *Trichuris* infective cycle starts with the ingestion of embryonated eggs, these eggs then travel to the caecum and within 90 minutes post ingestion L1 larvae emerge [13]. The larvae embed into the epithelial wall of the caecum, and after 4 moults and 35 days their posterior end is found extending into the lumen and eggs are released [2]. There is increasing evidence for interactions between *Trichuris* species and the host microbiota. First, interactions between bacteria and embryonated eggs are essential for hatching and secondly pre-treatment of mice with antibiotics prior to ingestion of eggs reduces the levels of infection [14]. The evolution of *Trichuris* species as successful pathogens of the large intestine would suggest that there is a long term adaptation of these parasites to exist in and interact with the commensal flora of the large intestine, and the development of the host immune system [12].

There have been conflicting results into the effect of *Trichuris* infections on the host’s intestinal microbiota [15–18]. Human studies have found that the presence of *Trichuris* species did not significantly impact on the microbial communities when looking in Ecuadorian individuals [15], however in infected Malaysian populations a difference in beta diversity and slight but significant increase in alpha diversity was observed [16]. In comparison, studies using porcine models found distinct changes in the relative abundances of different phyla and metabolic potentials of the microbiota [17,18]. Furthermore, given the widespread use of antihelmintic drugs to clear parasites in both the human, agricultural and domestic animal population, the effect of such drugs on the essential intestinal microbiota requires examination. Recently, other studies have looked at the therapeutic potential of clinical *Trichuris* infections to alleviate the symptoms of inflammatory diseases of the bowel which have had some success [19,20]. With this limited but conflicting information from human and animal studies, a detailed and controlled laboratory experiment was required to address the impact of chronic *T*.*muris* infections on the host caecal microbiota. In this paper we describe such a study in which a time course experiment was undertaken to monitor both the changes in intestinal microbiota in mice from egg ingestion over a period of 91 days and the effects on the microbiota of pathogen clearance. At the same time key immune cell populations associated with chronic *T*.*muris* infection (Th1 cells and FoxP3 T regulatory CD4+ T cells) were also analysed in the intestinal epithelium and lamina propria of the large intestine. Our results demonstrate that *T*. *muris* infection results in dramatic changes in host microbiota and perturbation of the metabolome of the large intestine. Upon pathogen clearance these changes were not maintained with a transition to a non-infected microbiota by the end of the experiment.

## Results

### Community profiling by DGGE

**Fig 1:**
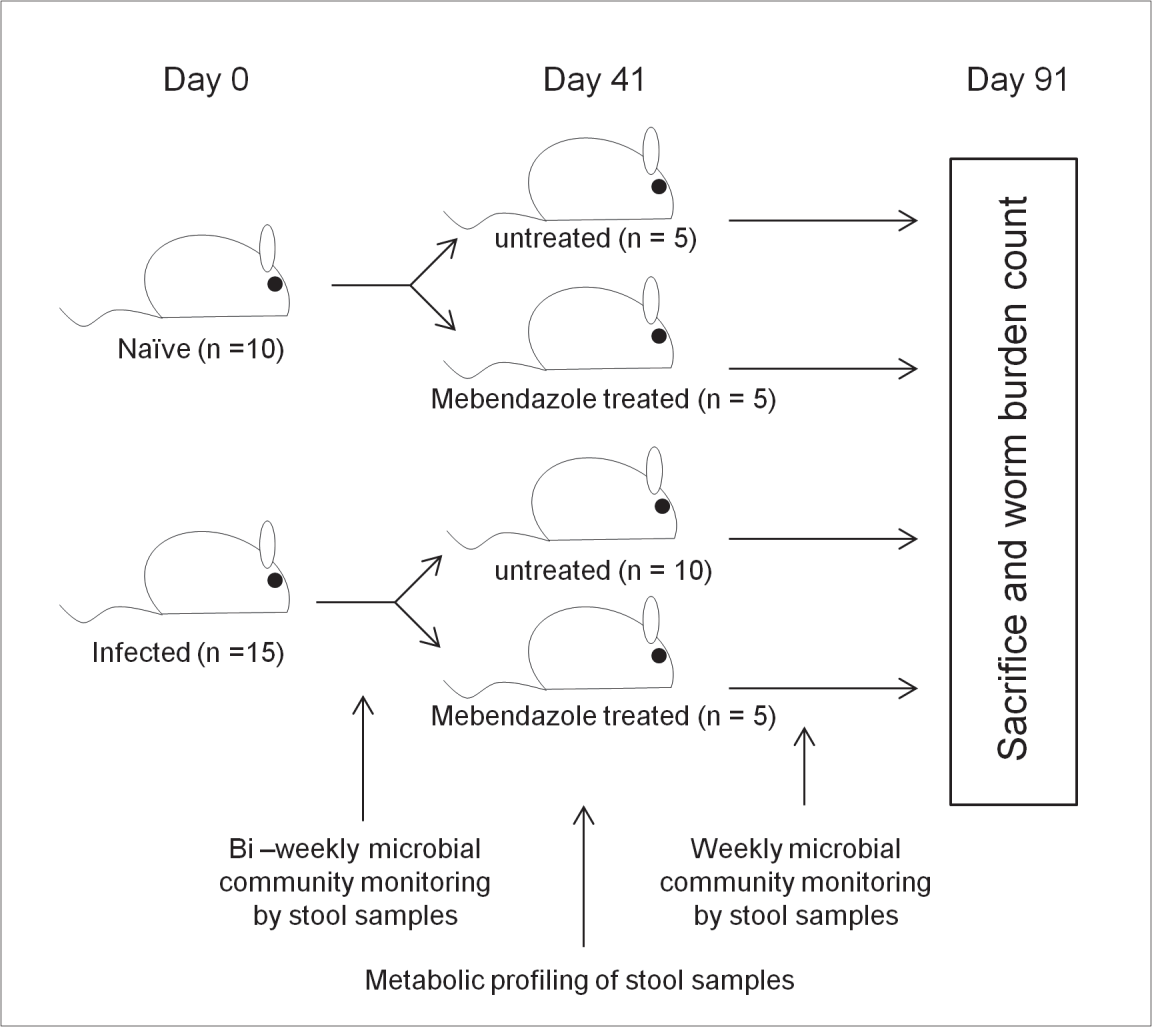
Experimental design: Sample timepoints and treatments for monitoring the intestinal microbiota during and after drug clearance of *T*.*muris* infection.

**Fig 2:**
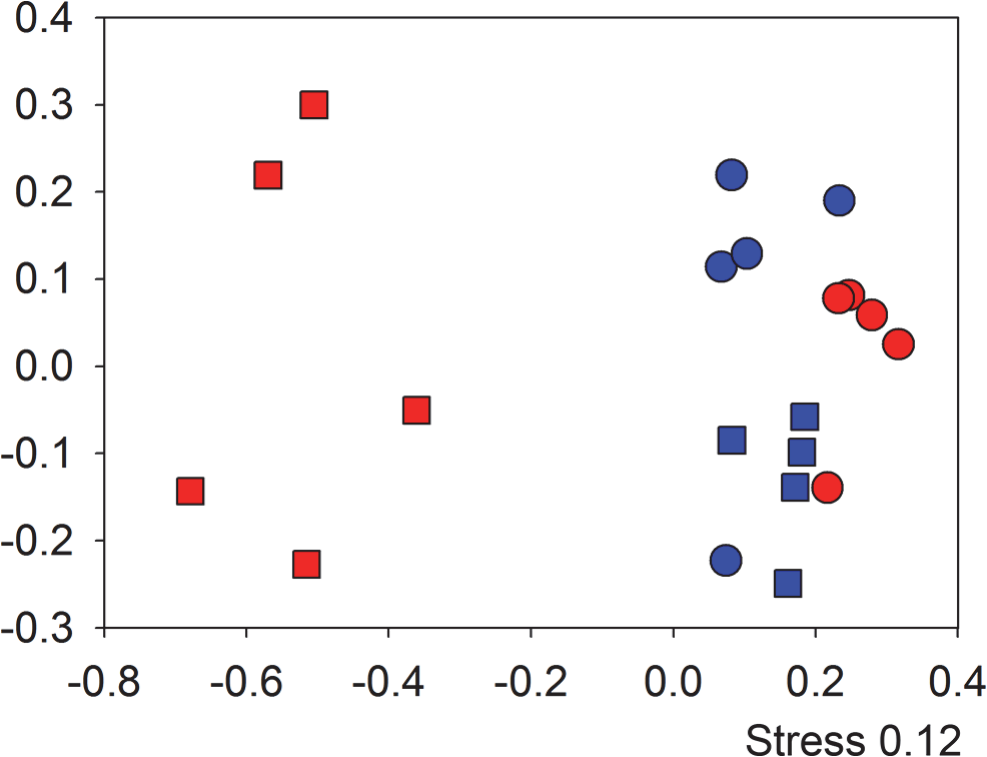
Impact of *T*.*muris* infection on microbial communities assessed by DGGE: NMDS analysis of bacterial community profiling in stool samples by 16S rRNA gene DGGE comparing day 0 to day 41 (○ & ◻) in infected (red) and non infected (blue) individual mice. Demonstrating a significant shift as a result of time and infection (F_1,16_ = 9.05, p = 0.002). Axis represent scale for Euclidian distance between samples centred on zero, Stress indicates the quality of fit of data (>0.2 is a good fit).

To study the impact on intestinal microbial communities during *T*.*muris* infections over time and to establish whether the changes observed were permanent or could be resolved following clearance of the *T*. *muris*, stool samples were collected and the microbiota assessed over a period of 91 days (Fig 1). Previous experiments (unpublished) on the caecal microbiota of *T*. *muris* infected C57BL/6 mice identified a significant change in the microbial communities in the caecum of mice at day 35 post infection, an effect that was maintained at day 72 post infection (S1 Fig). In this new study, a more detailed time course analysis was undertaken (Fig 1). A non-parametric multidimensional scaling analysis (NMDS) of DGGE data demonstrated the microbiota was stable at day 0 and day 14 with no treatment effect identified. At day 28 an initial shift was identified in infected mice stool samples and this increased by day 41 (S2 Fig). This clear shift in microbial communities of infected mice by day 41 in comparison to communities of naïve mice at day 0 and 41 (Fig 2) was analysed using PERMANOVA on the distance matrix created in NMDS analysis (Adonis function in vegan package R [21]) time (F_1,16_ = 7.06, p = 0.012) and infection (F_1,16_ = 5.99, p = 0.008) both had a significant effect, with the time ~ infection interaction giving the greatest effect (F_1,16_ = 9.05, p = 0.002). Analysis of the IgG2a/c response at day 35 confirmed that a successful chronic infection had been established (S3 Fig). Overall these data confirmed the results we have seen previously in caecal samples (S1 Fig) namely that by day 41 following infection there is a significant shift in the microbiota of the large intestine as identifiable in stool samples.

**Fig 3:**
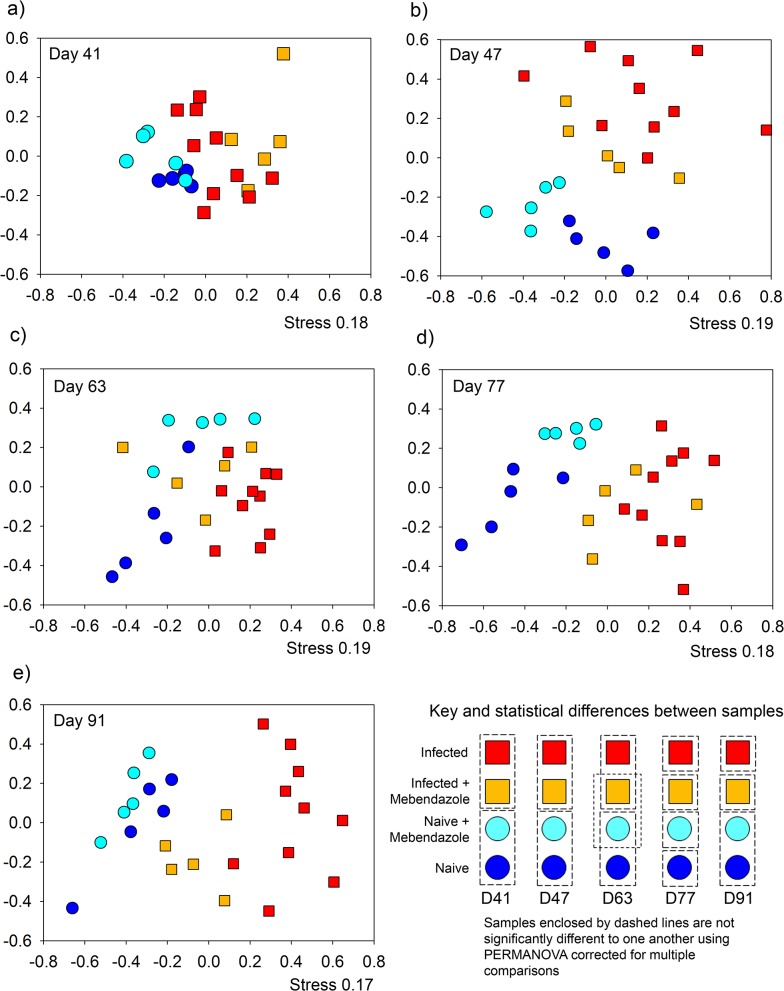
Time course monitoring of microbial communities by DGGE after antihelmintic treatment to clear infection: NMDS plots of DGGE analysis of bacterial communities of the 16S RNA gene in stool samples. Monitoring the change over time as a result of prolonged infection and clearance by antihelmithic treatment at a) day 41, b) day 47, c) day 63, d) day 77, e) day 91, f) The pairwise comparisons of treatments for significant differences assessed by PERMANOVA with bonferroni corrected p values < 0.0083. Axis represent scale for Euclidian distance between samples centred on zero, Stress indicates the quality of fit of data (>0.2 is a good fit).

To assess if infection by *T*.*muris* resulted in permanent or transient changes in the microbiota a subset of infected mice (n = 5) were treated with the antihelmintic, mebendazole from day 41 to day 43. To determine any effects of mebendazole treatment on the microbiota a control group of naïve mice (n = 5) were also treated with mebendazole (Fig 1). The monitoring of changes in the microbial populations by DGGE was undertaken weekly post mebendazole treatment. The data presented in Fig 3 is taken from alternate weeks for the period of the experiment. As six pair wise comparisons were undertaken on these data, a Bonferroni-adjusted significance level of 0.00833 was calculated to account for the increased possibility of type-I error (see S1 Table). At day 41, the time point of initiation of mebendazole treatment there was no difference between the naive and naive mebendazole treated mice. Likewise there was no significant difference between the infected and infected mebendazole treated mice. However both naive groups were significantly different to both infected groups (Fig 3A). At day 47 (Fig 3B) the relationships between groups was the same as day 41. This demonstrated that at this time point there was no detectable impact of mebendazole treatment on the microbiota of naïve mice and no detectable change in the microbiota of infected mice as result of clearing the infection. By day 63 (Fig 3C) the microbiota of naive and naive mebendazole treated mice were still clustered together as were the microbiota of infected and infected mebendazole treated mice. However a shift in the microbiota of the infected mebendazole treated mice resulted in it no longer being significantly different to the microbiota of the naive mebendazole treated mice (p = 0.06), indicating the beginnings of a shift in the microbiota of the infected mebendazole treated mice. At day 77 (Fig 3D) all treatments were significantly different to one another, however significance between the microbiota of naive, naive mebendazole treated and infected mice ranged from p = 0.0080 to p = 0.0081 (S1 Table) close to corrected significance level of 0.0083, whereas infected versus the other treatments ranged from p = 0.0004 to p = 0.0003 (S1 Table). By day 91 (Fig 3E) the microbiota of the infected mebendazole treated mice maintained a significant difference from the microbiota of infected mice and the microbiota of the naive and naive mebendazole treated mice, indicating that the microbiota of the naive, naive mebendazole treated and infected mebendazole treated mice were more closely related to one another than to the infected group.

Stochastic variation in cages causing "cage effects" have caused problems with experiments of this nature [22] so the naïve mice microbial populations were checked for time dependent changes from the time of mebendazole treatment (day 43) to the end time point to identify any potential changes are a result of treatments and not stochastic variation (S4 Fig). No time dependent effects were found (F_1,23_ = 1.87, p = 0.06), demonstrating high stability in mouse microbiota in this experiment

Taken together these data demonstrate infection of mice by *T*.*muris* causes a long-term and sustained change in the microbial communities of the mouse compared to naïve populations. Mebendazole treatment on naïve mice caused no detectable impact on the microbiota, however the clearance of infection in infected mice resulted in a change in microbiota that resulted in the community becoming increasingly similar to that of uninfected mice and dissimilar to infected mice.

### Pyrosequencing of key time points

DGGE analysis allowed us to monitor global shifts in microbial community composition and the timings of changes as a result of infection and the recovery of communities from post-infection. To characterise these changes in bacterial species as a result of infection, 16S rRNA gene amplicon pyrosequencing was undertaken on individual mice stool samples. Key time points during the experiment were identified for further investigation; day 0 to establish the baseline starting populations, day 28, the point when the first identifiable change in bacterial community as a result of infection was identified, day 41, in the presence of adult worms and the point of mebendazole treatment to clear infection, and day 91, the final time point of the experiment.

A total of 3,036,198 raw sequences were generated which was trimmed and cleaned as detailed in material and methods [23,24] resulting in 3,017,234 sequences with an average of 37,715 sequences per sample, and a minimum 9,567 sequences per sample (Deposited at the European Nucleotide archive: PRJEB7724). A total of 3,810 chimeric sequences were detected and removed from the analysis. Rarefaction curves demonstrate that samples have been sequenced to a suitable depth with the curves plateauing (S5 Fig). The depth of sequencing was estimated using goods coverage estimate [25] indicated there was on average 99.74% coverage with a range of 98.86% to 99.99% of OTUs sequenced in each samples. To assess the global impact on the bacterial diversity as result of the infection/treatments a rarefied OTU table of 9,567 sequences (lowest sequencing depth) was created by repeated sub-sampling of the data set using Quantitative insights into microbial ecology (QIIME) scripts [26].

### Impact on bacterial diversity

**Fig 4:**
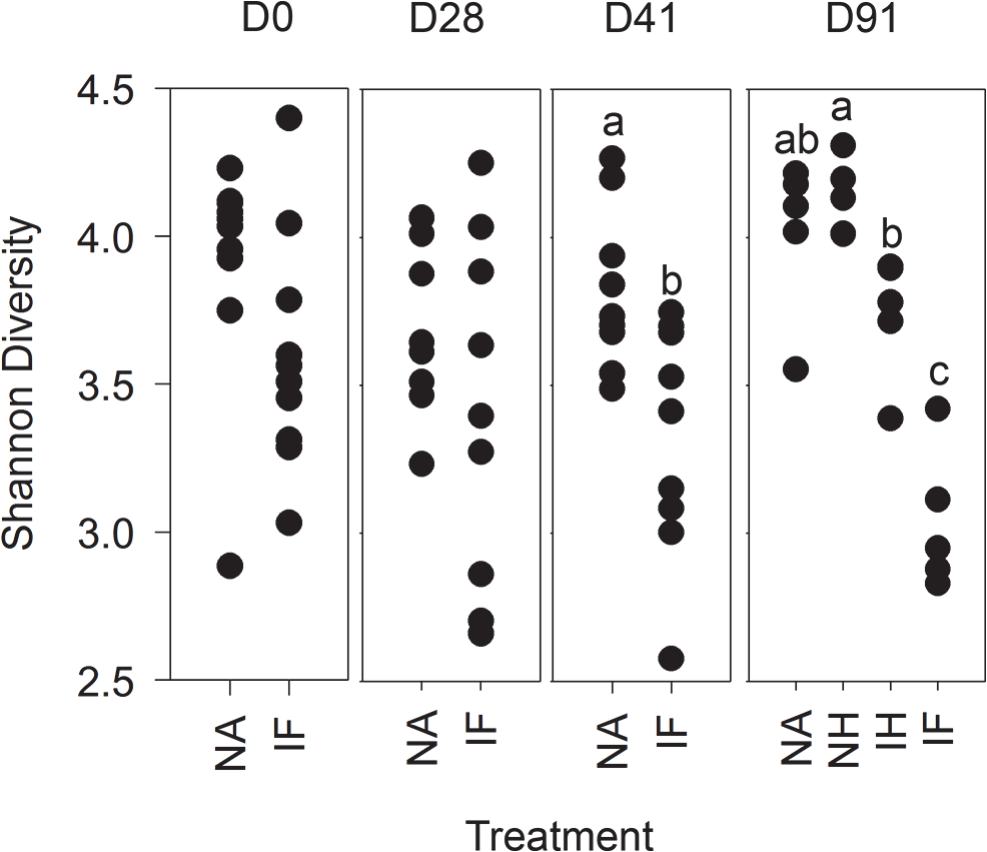
Shannon diversity from 454 pyrosequencing of stool samples over time: Microbial diversity was measured between treatments at each time point in individual stool samples from rarfied pyrosequecing OTU tables at 97% sequence similarity level. NA = naïve, NH naïve antihelminthic treated, IH = infected antihelminthic treated, IF = Infected. The labels a,b and c denoting samples significantly different tested by ANOVA (p = 0.05) with post hoc TukeyHSD tests as required.

Using the rarefied table, Shannon diversity (*S*) [27] was assessed in samples to identify global impacts on diversity as a result of infection (Fig 4). At day 0 the point of infection, as predicted, the diversity of the microbiota of naïve mice, *S* = 3.91 ± 0.12 s.e., was not different to that of the infected mice, *S* = 3.60 ± 0.12 s.e. At day 28 the result was similar with no significant difference between the diversity of the microbiota of naive mice, *S =* 3.75 ± 0.09 s.e. and infected mice *S* = 3.34 ± 0.19 s.e. However by day 41 the Shannon diversity of the microbiota of infected mice *S* = 3.32 ± 0.13 s.e. had significantly dropped in comparison to the microbiota of naïve mice *S* = 3.79 ± 0.09. For day 91, the samples had been split with 50% of each group treated with mebendazole for clearance of *T*.*muris* infection and drug treatment control at day 41. The mebendazole treatment had no effect on the microbial diversity in the naive mice, but had an effect on the infected mice, with mebendazole treatment resulting in an increase in diversity in the infected mebendazole treated group as compared to the infected group (Fig 4) as identified by post-hoc Tukey's HSD test on significant ANOVA result (F_3,16_ = 25.4, p < 0.001). In addition, the diversity of the microbiota in infected mebendazole treated mice was significantly lower than that in naive mebendazole treated mice. This indicated that by the end of the experiment the diversity of the microbiota of infected mice treated with mebendazole has increased from their infected state, but is still less diverse than the microbiota in uninfected mice. This demonstrates that following clearance of the parasite the microbial diversity recovers with time.

**Fig 5:**
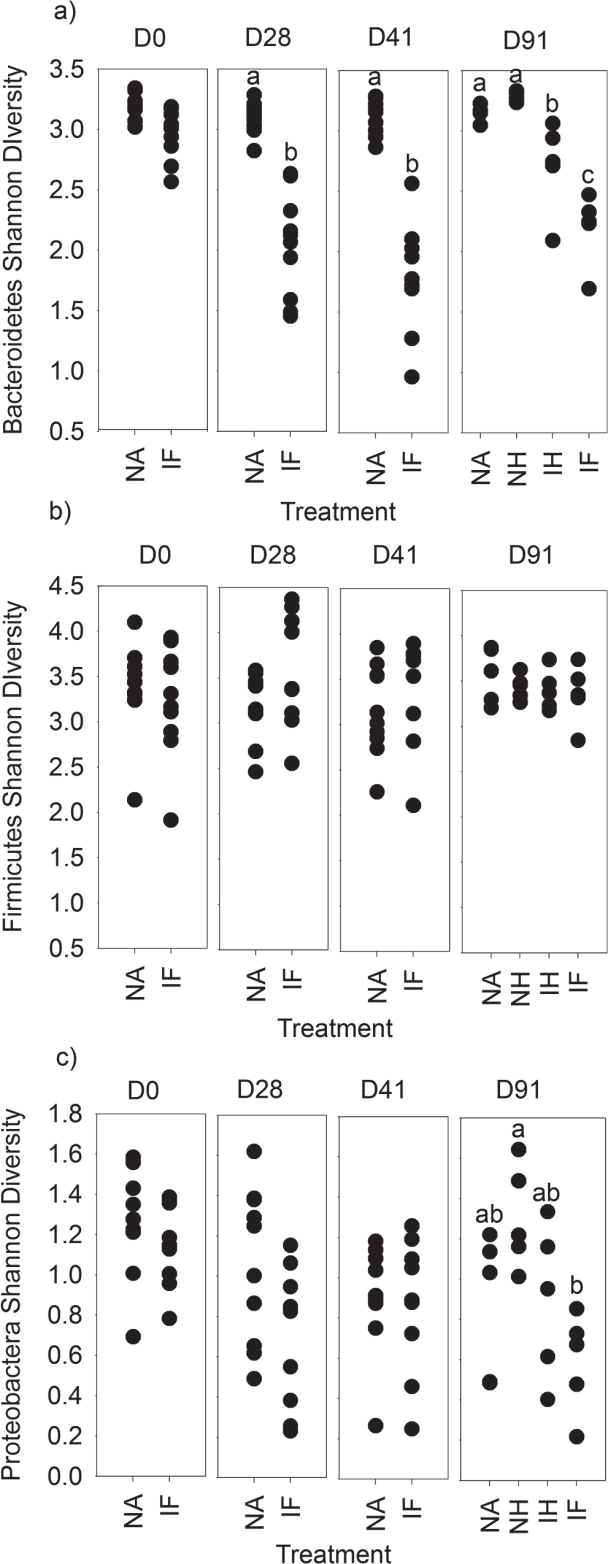
Shannon diversity of specific bacterial phyla in treatments from 454 pyrosequencing of stool samples: Microbial diversity was assessed for a) Bacteroidetes, b) Firmicutes, and c) Proteobacteria between treatments at each time point in individual stool samples from rarefied pyrosequecing OTU tables at 97% sequence similarity level. NA = naïve, NH naïve antihelmintic treated, IH = infected antihelmintic treated, IF = Infected. The labels a,b and c denoting samples significantly different tested by ANOVA (p = 0.05) with TukeyHSD tests as required.

Diversity of individuals was additionally assessed at the phyla level to identify if changes were general, or specific to particular phyla. Diversity comparisons were assessed on three dominant phyla from the stool samples (Fig 5). Analyses of the diversity of Bacteroidetes (Fig 5A) demonstrated that by day 28 the *T*. *muris* infection had significantly reduced the diversity of infected (2.03 ± 0.14s.e.) in comparison to naive (3.09 ± 0.04 s.e.). The difference in diversity increased at day 41 with naive and infected (3.12 ± 0.05 s.e. and 1.79 ± 0.16 s.e. respectively) significantly different to one another. At day 91 ANOVA identified a significant effect of treatment (F_3,16_ = 20.94, p << 0.001) on diversity with post-hoc Tukey's HSD test identifying that only naive and naive mebendazole treated were not significantly different to one another, whereas the diversity in the microbiota of infected mebendazole treated mice was significantly different to all other groups. In addition the microbiota diversity in infected mice was significantly lower than all other groups. This demonstrates that the Bacteroidetes are impacted by the *T*. *muris* infection and that removal of *T*. *muris* has an effect, beginning to restore Bacteroidetes diversity back to that of the uninfected animals. Assessment of Firmicutes (Fig 5B) demonstrated there was no treatment effect at any time point, indicating that there appears to be no impact on the diversity of Firmicutes as a result of infection. A similar story is seen when assessing the Proteobacteria diversity (Fig 5C) with no treatment effect at day 0, day 28, and day 41, with a small significant effect at day 91. Post-hoc Tukey's HSD test identified that this was a difference between the naive mebendazole treated (1.30 ± 0.11 s.e) and infected mebendazole treated (0.59 ± 0.11) mice.

### Community Composition analysis

**Fig 6:**
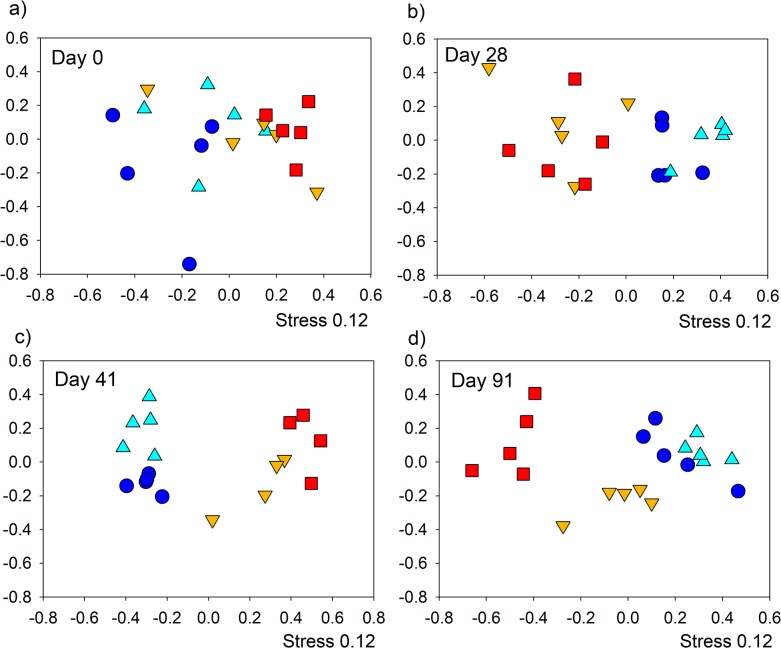
NMDS analysis of bacterial communities in stool samples assessed by 454 pyrosequencing at individual time-points: Comparisons were made between the groups Naïve, naïve antihelminthic treated, infected antihelminthic treated and Infected, in order of dark blue, light blue, orange, red. a) Day 0 there were no significant differences, b) day 28 & c) day 41, naïve and naïve antihelminthic treated were significantly different to infected and infected antihelminthic treated (p < 0.0083), D91 all significantly different (p < 0.0083). Axis represent scale for Euclidian distance between samples centred on zero, Stress indicates the quality of fit of data (>0.2 is a good fit).

Bacterial Community composition was assessed by NMDS on the community composition on the rarefied OTU table to prevent bias by sequencing depth. This allowed the comparison of the compositional make up of communities to be compared between samples, over time. NMDS analysis demonstrated a clear shift in the microbial communities as a result of *T*. *muris* infection from day 28 separating away from the naive communities with the indication of the infected mebendazole treated communities returning towards a naive population by day 91 after clearance of the infection (Fig 6). Each treatment/cage was compared at each time point with Bonferoni correction to p < 0.0083. At day 0 (Fig 6A) the baseline start point, there was no significant differences between samples. At day 28 (Fig 6B) there was clear separation of infected mice from naïve mice, this separation continued at day 41 (Fig 6C) between Naïve treatments and Infected treatments with an increase distance between groups on NMDS. By 91 (Fig 6D) all samples were significantly different to one another. No cage differences were identified within groups at any time points.

To identify if separation on NMDS plots correlated with proportion of bacterial families, vectors were plotted onto the NMDS in S6 Fig using Vegan package [21] in R that represent individual bacterial family proportions. The direction of the arrows indicate greatest gradient of change. Due to multiple vector plotting, p values of correlations were adjusted by False Discovery Rate (FDR) correction and corrected regressions were plotted (S6 Fig). Twenty families were identified to have changes that correlated with separation of microbial communities plotted by NMDS. These composed of an Actinobacteria, five Bacteroidetes, nine Firmicutes, three α-Proteobacteria, an uncultured TM7 family of bacteria, and a Defferibaceraceae (S6 Fig). One must be cautious in interpretation of these plots since they represent correlations, not actual changes in proportions as a result of treatment.

### Identifying significant shifts in bacterial proportions

We identified that there was no significant difference in bacterial densities in the caecum between infected and naïve mice when assessed by Q-PCR of the 16s rRNA gene (data not shown), therefore using the OTU table created with the pick_de_novo_otus.py workflow in the QIIME software [26] analysis was undertaken on shifts in bacterial proportions. Baseline populations of naïve mice at the start of the experiment had 3 predominate phyla present in stool samples, they consisted of Bacteroidetes (61.4% ± s.e. 5.1), Firmicutes (37.2% ± s.e 5.1) and the Proteobacteria (0.7% ± s.e 0.1) making up over 99% of sequences.

Bacterial community proportions at the family level were compared between naïve and infected individuals taking the average proportions at each time point (S7 Fig). This clearly demonstrates a shift in the microbial community proportions as result of infection with indications of changes in Bacteroidiaceae, Deferribacteriaceae, Lactobacillales, Odoribacteraceae, Prevotellaceae Rikenellaceae and the S24-7 group of uncultured Bacteroidetes as a result of infection several of which were shown in correlative analysis (S6 Fig).

**Fig 7:**
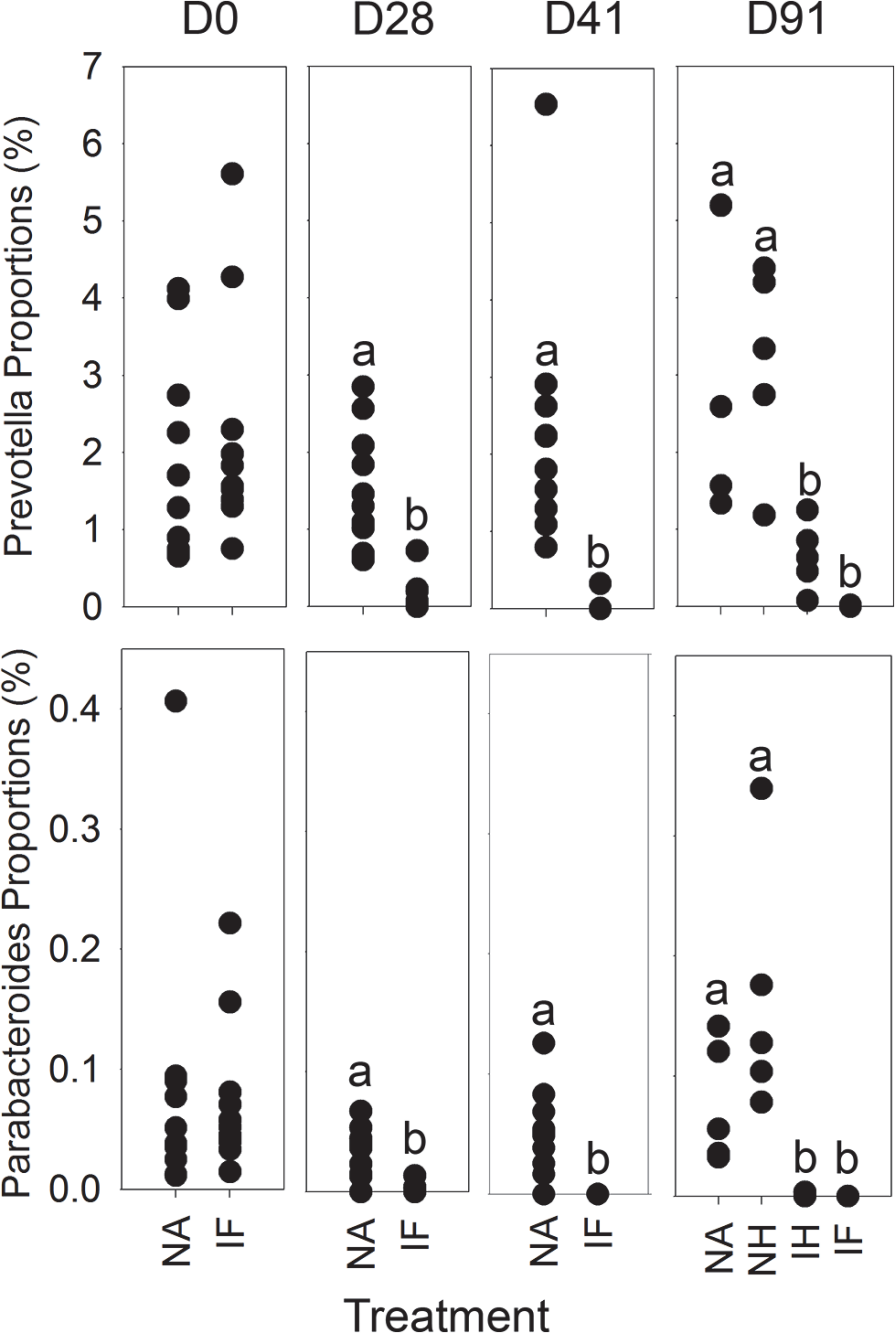
Impact of infection by *T*.*muris* on bacterial community composition: Bacterial community proportions of Prevotella and Parabacteroides were identified as significantly altered as a result of treatment in relation to time, and a level of recovery as a result of antihelmintic treatment of Prevotella populations. NA = naïve, NH naïve antihelmintic treated, IH = infected antihelmintic treated, IF = Infected. The labels a and b denoting samples significantly different tested by ANOVA (p = 0.05) with post hoc TukeyHSD tests as required.

To identify significant changes in microbiota proportions that are a result of treatments ANOVAs were undertaken on family/genus levels data to identify differences between treatments at each time point. Type I errors occur at the same frequency (5% at p = 0.05), therefore multiple comparisons increases the likelihood of such errors and were corrected using the FDR correction [28]. At day 0, there were no differences between the naive groups and the infected groups confirming the baseline starting populations were the same between groups. Two populations of bacteria demonstrate significant changes as a result of time and infection (Fig 7). Prevotella and Parabacteroides both demonstrated a significant reduction in their proportions of the microbiota as a result of the *T*. *muris* infection by day 28, (12 fold and 15 fold reduction respectively), this impact was increased by day 41 with a 70 fold reduction for Prevotella, and dropping to below detection level in Parabacteroides. By day 91 following mebendazole treatment at day 41, the Prevotella populations demonstrated a similar result to that seen at day 28 and day 41 with Prevotella proportions significantly higher in naive and naive mebendazole treated in comparison to infected mebendazole treated and infected (Fig 7). However by day 91 there appears to be the beginnings of recovery from the infected state in the infected mebendazole treated mice, with their Prevotella populations moving towards that of the naive and naive mebendazole treated (Fig 7). In the Parabacteroides the results indicate populations in the infected mebendazole treated and infected samples have remained low and were not significantly different to each other. Likewise the naive and naive mebendazole treated groups are not significantly different to one another (Fig 7). Other bacterial genera that have changes identified at specific time points include: at day 28 *Mucispirillum* species which significantly increase from 0.3% ± 0.09 to 1.5% ± 0.3 (5 fold increase, p.adjusted = 0.048), and at day 41 there was a significant increase in Rikenellaceae from 3.7% ± 0.7 to 9.5% ± 0.2 (2.6 fold increase, p.adjusted = 0.045), and family F16 from the uncultured TM7 phyla 0.02% ± 0.005 to 0.18% ± 0.05 (9 fold increase, p.adjusted = 0.01) and at day 91 a number of small community changes were identified between groups detailed in S2 Table.

### Core microbial communities

To investigate if the core microbiota changes during the course of the experiment, differences in OTU numbers from the major phyla detected in mice were examined from the OTU table created in QIIME. At day 28 there were 43 Bacteroidetes OTUs, 19 Firmicutes OTUs and 2 Proteobacteria OTUs conserved in naive mice. However infection caused this to change and the conserved microbiota in infected mice consisted of 12 Bacteroidetes OTUs, and 22 Firmicutes OTUs. This demonstrated a shift from Bacteroidetes to Firmicutes in the conserved core microbiota present as a result of infection. This shift was more pronounced by day 41 with 41 Bacteroidetes OTUs, and 15 Firmicutes OTUs conserved in naive mice compared to 5 Bacteroidetes OTUs, 38 Firmicutes OTUs and 2 Proteobacteria OTUs conserved in infected mice. This demonstrates a shift in conserved OTUs in stool samples from predominantly consisting of Bacteroidetes in naïve animals to being predominated by Firmicutes in infected animals which increases with time. This does not impact on diversity or proportions of Firmicutes, but may reflect the decreased diversity and stability of the Bacteroidetes communities, and an increased stability of the Firmicutes populations.

### Metabolic difference with infected and naive mice at day 41

**Fig 8:**
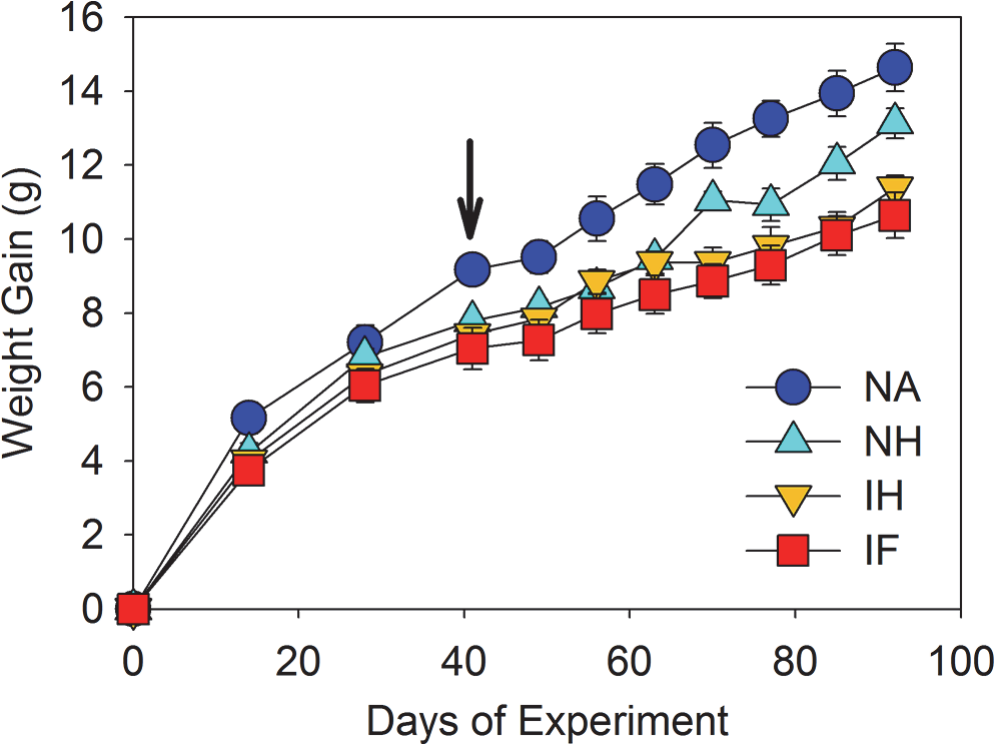
Impact of *T*. *muris* infection of mouse growth: At day 91 there is a treatment impact (F_3,21_ = 9.04, p <0.001) which TukeyHSD tests identified as naïve gaining more weight than infected and infected antihelmintic treated (p <0.001 and 0.01 respectively). Naive antihelmintic treated were not different to naïve, but significantly higher than infected (p = 0.03). NA = naïve, NH naïve antihelmintic treated, IH = infected antihelmintic treated, IF = Infected. Arrow indicates point of antihelmintic treatment.

To identify if changes in the microbiota as a consequence of infection resulted in changes in the metabolic profile in the large intestine, stool samples were taken from mice at day 41 and a comparison was undertaken between the naïve (n = 10) and infected (n = 10) mice using GC-MS and LC-MS (± and-) analysis. NMDS analysis of metabolic profiles identified very distinct impact of infection (S8 Fig). GC-MS analysis identified an increase in a number of amino acids as a result of infection; Phenylalanine (51.23 fold increase, p.adjust = 0.03), Serine (17.39 fold increase, p.adjust = 0.03), Thereonine (9.41 fold increase, p.adjust = 0.03), Leucine (8.89 fold increase, p.adjust = 0.02), Ornithine (7.68 fold increase, p.adjust = 0.03), and Glycine (2.7 fold increase, p.adjust = 0.03). LC-MS analysis identified a decrease in a large number of metabolites (S3 Table). Analysis of these data identified a significant reduction in derivatives of Vitamin D2/D3, a large number and range of fatty acids and related metabolites, and glycerophospholipids (S3 Table). There was also a reduction in breakdown products of dietary plant derived carbohydrates and intermediates involved in amino acid synthesis, such as the biosynthesis of Phenylalanine, Tyrosine, and Tryptophan, Lysine, Cystine and Arginine, and reduction in the detection of breakdown products of Heme (S3 Table). To establish if such metabolic changes reflected the ability of the mice to maximise nutrient release from their diet the mass of the mice was determined throughout the experiment (Fig 8). At day 91 there is a treatment impact (p <0.001) which TukeyHSD tests identified as naïve gaining more weight than infected and infected mebendazole treated (p <0.001 and 0.01 respectively) and likewise the naive mebendazole treated mice (not different to Naïve mice) had increased in mass more than infected mice (Fig 8). This indicates that infection causes a small but significant reduction in the weight gain of the mice over the course of the experiment, with the infected antihelmintic treated not showing any level of recovery. We would suggest that this is a consequence of changes in the microbiota which have been shown by the end of the experiment to not have fully recovered and thus may impact on the ability of the microbiota to process dietary food stuffs and recover weight.

### Impact of infection and clearance on immune responses

Key immune cell populations associated with chronic *T*. *muris* infection (T-bet± Th1 cells and FoxP3± regulatory CD4± T cells) were analysed in the intestinal epithelium and lamina propria of the large intestine on day 41, prior to antihelmintic treatment and again at the conclusion of the experiment on day 91. There were clear changes associated with long-term infection including a reduction in % FoxP3± regulatory CD4± T cells within the lamina propria and % T-bet± Th1 cells in the epithelium. Interestingly, antihelmintic treatment of infected mice, and the associated recovery of the microbiota, failed to restore the FoxP3± regulatory CD4± T cell population within the lamina propria, but did significantly restore the T-bet±Th1 cell population within the epithelial compartment (S9 and S10 Figs). This would indicate that changes in the intestinal microbiota in response to parasite infection are linked with changes in some specific immune cell types, however some immune responses remain unaltered.

## Discussion

The microbiota in the intestinal tract play important roles in health and nutrition, with perturbations causing disease and sometimes extended dysfunction of the gut. Analysis of the effect of *Trichuris* on the microbiota of the large intestine has only recently been investigated in endemic human populations infected with *T*. *trichiura* or experimentally *T*. *suis* infected pigs with variable and sometimes conflicting results [15–18, 20]. We undertook a detailed and well-controlled study to assess perturbations in the microbiota throughout chronic infection with repeated sampling of individual mice. We have identified significant impact on the host microbiota as a result of helminth infection in C57BL/6 mice. Time course analysis demonstrated that the β-diversity of stool microbiota assessed by NMDS of DGGE changes as a result of *T*. *muris* infection between day 14 and day 28 (S2 Fig), this significant impact on the β-diversity was confirmed with the deep sequencing of 16S rRNA gene amplicons of stool samples at day 0, day 28, and day 41 (Fig 6), highlighting a change in the microbiota when *T*. *muris* is at the L3- L4 stage [4]. Studies by Li *et*. *al*. also identified changes in the microbiota by day 21 in the porcine colon [17] which occurs at a similar larval stage to our study [29].The difference in the β-diversity increase by day 41 indicated by both DGGE, and sequencing (Fig 6 & S2 Fig), confirms the impact seen in preliminary experiments assessing changes in caecal microbiota (S1 Fig). Assessment of the α-diversity using Shannon diversity index [27] identified that there is a significant reduction in diversity as a result of infection (Fig 4) which was apparent at day 28, but became significant by day 41. These data are the first to demonstrate marked reduction in α-diversity as a result of *Trichuris* species infection. In humans it had been shown that helminth infection caused a very small increase in diversity [16], however the individuals had mixed helminth infections including *Necator americanus* and *Ascaris spp*. making unequivocal assignments of cause and effect difficult. In a human study where *N*. *americanus* was the only soil-transmitted helminth, however, no significant differences in microbiota were found [30]. There may be a number of potential reasons for these observed differences between these different experiments. In part it could reflect that different parasites were studied including mixed infection Likewise differences in sample preparation and DNA amplification for sequencing together with subsequent sequence analysis and reference data base selection could also account for some of these differences. Infection of pigs by *T*. *suis* had no significant effect on α-diversity at day 21 on the microbiota [17]. *T*. suis is present as a larval stage at this point, and the data presented here would indicate that significant changes in total bacterial diversity do not occur until later, e.g. at patency at day 41.

We identified that the predominant three phyla in the naive mouse microbiota were Bacteroidetes (61.4% ± 5.1 s.e.), Firmicutes (37.2% ± 5.1 s.e.) and the Proteobacteria (0.7% ± 0.1 s.e.) whose proportions are very similar to previously described in C57BL/6 mice [31], and thus we assessed the α-diversity of these groups to identify if infection has a global impact or phyla-specific impact. Infection caused a Bacteroidetes-specific decline in diversity (Fig 5), which was detectable at day 28 in comparison to the reduction in total bacterial diversity detectable at day 41. Interestingly there appears to be no impact on the Firmicutes or Proteobacteria diversity demonstrating high stability (Fig 5).

Bacteroidetes are very important bacteria in the intestine and are regarded a specialist in the degradation of carbohydrates having the ability to utilise an extensive range of substrates [32]. They are important in the breakdown of high fibrous plant derived diets, and often are enriched in comparison to Firmicutes in consumers of non-western diets [33]. The anaerobic breakdown of carbohydrates by Bacteroidetes fermentation releases short chain fatty acids (SCFA) which are easily absorbed by the host and allow the host to gain energy from otherwise inaccessible sources and changes in the levels of SCFA in the intestine can change the pH in the lumen and impact upon the composition of the microbiota present [34]. The mice under experimentation are fed a very high fibre plant derived diet consisting of feed made from Wheat, Barley, Soya, and Maize (15% total fibre) and thus reduction in Bacteroidetes diversity and numbers may impact on intestinal function in the mice and impact on health. Thus we investigated the intestinal metabolome to identify changes that result from the parasite infection. Any potential changes could result from changes in host absorption, alterations in the microbial metabolome and the release of metabolites from *T*. *muris*. The metabolomic data identified a reduction in plant-derived metabolites, fatty acids and related metabolites in stool samples, which would indicate that there may be a reduction in the degradation of plant derived carbohydrates in the intestine. This would impact on the nutrient uptake by the mice that would be reflected in the reduced weight gain in the infected mice during the course of the experiment. A large number of other metabolomic changes were observed in the stool samples as a consequence of infection with the increase in the number of essential amino acids being particularly curious (S3 Table) These responses appear to be different to those seen in gut inflammation models and human colitis studies which have demonstrated reductions in amino acids and related metabolites as a result of inflammation [35–37]. This may not be surprising as chronic low dose *T*. muris infection does not result in overt colitis but a regulated intestinal response, which is under the control of Interleukin 10 [38–39]. Furthermore, bacterial derived SCFA have recently been identified as key modulators of the intestinal immune system, and can directly induce Foxp3± regulatory Tregs in the colon [40]. Interestingly, infected mice, as well as demonstrating a reduction in the bacteria which produce SCFAs also demonstrated a significant decrease in the Foxp3± Treg population during long-term infection in the lamina propria (S8 Fig). At this stage it is impossible to predict the origins of such metabolomic changes and the biological consequences. For instance whether they are direct result of changes in the host microbiota or a direct consequence of the metabolism of the parasite is as yet unclear. It is perfectly possible that it is the release of metabolic by products from *T*. *muris* that directly affects the composition of the host microbiota.

Correlative vector analysis of family proportions onto NMDS plots (S6 Fig) identified twenty bacterial families that demonstrated shifts which appeared to have links with separation in NMDS as a result of treatments indicating a large general shift in communities as a result of infection. Comparisons between samples to identify the proportions of bacterial genera/families that had significant shifts as a result of treatment, identified that there was a significant impact on the Prevotella and Parabacteroides populations as a result of infection (Fig 7), both being reduced as a result of infection, and both belonging to the Bacteroidetes which contribute to the drop in diversity in this phyla. This observation was also seen in human studies with reduction in Prevotellaceace species as a result of *Trichuris* infection in Malaysian populations [16]. Prevotella has been demonstrated to be enriched in high fibre diets, whereas an increase in the Bacteroidetes has been found where there is also high fat in the diet [41]. The reduction in Prevotella may influence proportion of plant metabolites and related products that have been detected in the stool samples of infected individuals demonstrating reduced digestion of feed. This may explain the slight but significant reduction in weight gain of mice that are infected (see above). We also identified at day 28 *Mucispirillum* species which significantly increased at this time point only. These bacteria colonise the mucin layer of the gut [42], and have previously been demonstrated to be impacted on by *Trichuris* infection at a very similar time point in pigs [17]. During infection by *Trichuris* marked changes in epithelial cell turnover [2], goblet hyperplasia [43] and mucin production/glycosylation are observed [44]. This would result in markedly altered mucin secretion potentially providing a favourable habitat for the *Mucispirillum* species. Interestingly, increased levels of serine and threonine were seen in stool samples after infection, which are major components of mucins [45] which may result from mucin degradation in the gut, possibly by via *Trichuris* secretions [44]. The recently described regulator NLRP6 has been demonstrated to be involved in the regulation of faecal microbiota [46] and has been demonstrated to regulate and influence goblet cell proliferation and mucin secretions [47] which are observed following infection [43]. Levels of NLRP6 have been inversely correlated to the levels of Prevotellaceace in the gut [46], thus increased NLRP6 expression resulting in the changes in the mucin secretion that we see during infection, may be a factor in the reduction of Prevotella that we identified. NLRP6 is also a leucine rich protein which may contribute to the increase in leucine found in stool samples.

The effects of infection on the Bacteroidetes is apparent when looking at the conserved OTU data between the mice in different groups. As the infection progressed there is a shift from a predominance of Bacteroidetes to Firmicute OTUs such that by day 41 infected mice had only 5 Bacteroidetes, but with 38 Firmicutes conserved between them. This does not impact on diversity of Firmicutes, but may reflect the decreased diversity and stability of the Bacteroidetes communities who dominate the uninfected gut such that their decrease during infection allows an increased stability in the Firmicutes.

Our findings on the effects of infection on the microbiota are in keeping with the results presented in the companion study by Holm *et*. *al*. We both observed similar changes despite experiments being undertaken in different countries with different experimental approaches. Both studies clearly identified a significant change in bacterial communities as result of infection between day 14 and day 28 by NMDS analysis and a reduction in total bacterial Shannon diversity by day 28, with major reductions of Bacteroidetes groups.

To assess if the perturbation in the host microbiota following infection was short term or persisted once the pathogen was removed, analysis was undertaken following removal of the worm burden. Control naïve mice treated with mebendazole exhibited no significant impact on their microbiota over the course of the experiment. Infected mice treated with mebendazole to clear infection (clearance of worms occurs within seven days following treatment) demonstrated a slow transition in their microbiota, which over the 50 day period subsequent to treatment became markedly different to the non treated infected group, with increasing similarity to the naïve populations (Fig 3). This result was confirmed by the sequencing data at day 91 (Fig 6), with the shift of communities away from infected populations. Analysis of α-diversity demonstrated the same change with an increase in the diversity in treated mice, although this had not returned to naïve population levels (Fig 4). Recovery in α- diversity in Bacteroidetes was clearly identified with a significant increase in the treated individuals, but not enough to return to the naive populations by the termination of the experiment. Proportional data identifying the Prevotella and Parabacteroides demonstrated a significant drop in these populations as a result of the infection, with clearance demonstrating signs of recovery in the Prevotella, although this was not significant. Less of an impact was seen in the Parabacteroides which may be a result of populations dropping below detection level and as they represented a very small percentage of the community, may have been completely lost in some samples. These data are the first we believe to show significant changes of the microbiota as a result of the clearance of *Trichuris* infection under controlled conditions. The microbiota have not fully returned to their original state, potentially a result of the large and significant impact of infection on the microbiome taking a long time to recover, in addition to the damage to the intestinal epithelia. We also identified some species that dropped below detection level which may have been permanently lost, and interestingly that there was a persisting decrease in the proportions of the FoxP3± CD4± T cells despite infection clearance that could impact on the selection pressures on the intestinal microbiota. Recent studies on the effects on the microbiota of infected humans treated with an antihelmintic failed to detect any effects [15]. However samples were taken 21 days post treatment, which according to our data is likely to be too soon to see any effects of helminth clearance on the host microbiota. Human studies on indigenous populations are notoriously challenging with multiple compounding effects impacting on microbiota analysis even after helminth clearance

To summarise we identified significant changes that occurred between 14 and 28 days post infection in the microbiota, which then persisted to the termination of experiments after 91 days. There were significant changes in the α and β- diversity of infected mice, predominantly affecting the diversity and abundance of Bacteroidetes, specifically Prevotella and Parabacteroides. These changes in communities were linked with changes seen in the metabolomics of stool samples of mice indicating reduction in plant derived carbohydrates metabolism and bi-products, and immune and signalling responses that may have resulted in the increase in amino acids in the stool samples. The clearance of infection demonstrated for the first time to our knowledge a level of recovery of intestinal microbiota post infection, an observation that had not been recorded previously [15]. This recovery indicates that once the parasite is removed the selective pressure to maintain the “infected” microbiota is lost with evidence of recovery back to an uninfected microbiota with time. Changes in populations of key T helper cell subsets in the intestine were also observed accompanying long-term infection although modest in nature. Of most interest was the association between the removal of the parasites, recovery of a more “naïve” flora and recovery in the Th1 cell population in the epithelial compartment, to levels similar to that originally seen in uninfected animals, Whether these changes are functionally related remain to be determined. It is also interesting to note that the proportion of FoxP3± CD4± T cells within the lamina propria remained significantly lower than naïve animals even though parasites had been eliminated over a month earlier. This reflects the highly regulated chronic response to *T*. *muris* [39] suggesting long lasting immunological consequences of dysbiosis. In non-laboratory conditions in which the individual may be exposed to continual, multiple, and different sources of infections, these dysbioses in the microbiota, metabolome and immunology may have implications in terms of both susceptibility and long-term pathology.

## Materials and Methods

### Animals and sampling

Male C57BL/6 mice (6–8 weeks) were kept at 22°C ± 1°C and 65% humidity with a 12 h light-dark cycle and had free access to food and water. Animals were housed in individually ventilated cages in groups of 5 with diagnostic ear punches to identify individuals. All animal procedures were performed according to the UK Animals (Scientific Procedures) Act (1986) with the animals being monitored on a daily basis. During the course of the experiments we had no unexplained mouse fatalities. In addition during the experiments none of the mice showed any overts signs of distress for example, weight loss, poor body skin or fur condition. Mice were humanely killed by CO_2_ inhalation followed by cervical dislocation or terminal exsanguination.

Mice were sourced from Harlan, UK, and were from the same batch to control for between batch difference in the mouse GI tract microbiota (previously seen in unpublished experiments). Mice were housed in the facility for 2 weeks prior to experiment to stabilize communities to new conditions. Mice were infected by oral gavage with *T*. *muris* strain E for a low dose infections using ~ 20 eggs and parasite specific antibody levels in the sera were measured as described [48] to confirm infection. To assess impact of *T*. *muris* infection on the GI tract in mice, C57BL/6 mice were used to monitor microbial changes in stool samples as a result of chronic low dose infections and clearance over a period of 91 days (Fig 1). Mice were split into 4 groups, Naive, Naive treated with the anti-helminth mebendazole at day 41–42 (dose 50mg kg^-1^:drug treatment control), Infected, and Infected treated with mebendazole at day 41–42 to clear infection. Confirmation of chronic infection was undertaken by parasite specific sera IgG2a/c ELISA reflecting a Th1 response (detailed below). Efficacy of antihelmintic treatment was confirmed by negative faecal egg counts after treatment (typically 7 days after treatment) and intestinal worm burden at the termination of the experiment.

### Sample collection

Fresh stool samples were collected at 9:30 am from individual mice on sampling days to allow monitoring of individual mice and limit diurnal effects. This was undertaken every 2 weeks until day 41, and then weekly until the termination of the experiment from individual mice to allow monitoring of changes in bacterial microbiota in individuals over time (Fig 1). Samples were stored at -80°C until DNA extraction and metabolomic anaysis.

### DNA extraction and community profiling

Genomic DNA was extracted directly from stool samples using the QIAamp DNA Stool Mini Kit (Qiagen) using the with pathogen protocol. Community profiling of bacterial communities in stool samples was undertaken by Denaturing Gradient Gel Electrophoresis (DGGE) and 454 sequencing (Roche, USA). DGGE assessment of the bacterial communities was as follows: PCR amplification of the 16S rRNA gene used universal primers 341F-GC and 518R [49], and reaction conditions: 5U BioTaq polymerase in 1X buffer (Bioline, UK), 1.5 mM MgCl2, 20 pmol primers, 0.2 mM dNTPs, 5 μg BSA, and 10–50 ng of template DNA in a final volume of 50 μL. The cycle sequence consisted of initial dentaturation step of 95°C 5 min, then 30 cycles of 95°C 1 min; 55°C 1 min; 72°C 1 min, and final extension of 72°C 10 min. PCR products were purified (QiaGEN Minelute kit) before loading onto a DGGE gel (200 ng / lane). Sample were separated using the D-code system (Bio-Rad, USA) on 10% w/v acrylamide gel with a gradient of 30–70% denaturant at 60°C for 16 h at 63 V. Gels were stained for 30 min using SYBR Gold (Invitrogen, USA). DGGE Gels were analyzed with Phoretix 1D Advanced gel analysis software (Ver. 5.0, Nonlinear Dynamics Ltd.), with binary matrix of band presence/absence of individual bands used for sample comparison.

454 sequencing of the bacterial communities were as follows: The 16S rRNA gene was amplified using the modified 16S primers 66f and 518R (italicised) that cover the V1 to V3 region and include the Lib-A linker primer sequences required for 454 sequencing and a MID tag to allow sample pooling. (Forward primer (Primer A-Key): 5’‐CGTATCGCCTCCCTCGCGCCATCAG(MID)*CAGGCCTAACACATGCAAGTC*‐3’

Reverse primer (Primer B-Key): 5’‐CTATGCGCCTTGCCAGCCCGCTCAG*ATTACCGCGGCTGCTGG*‐3’

Roche multiplex identifiers (MID tags), which are unique "barcode" sequences for each amplified sample were used to allow the pooling of different samples into the same sequencing run. Post sequencing these samples could then be separated on their MIDs back into the individual sample amplified for analysis. All samples were amplified by PCR using the same batch of reagents/buffers to eliminate reagent difference effects, they were also amplified in triplicate to reduce variation in PCR amplicon products [50]. Samples were amplified using the following reaction conditions: 3U Velocity polymerase in 1X buffer (Bioline, UK), 20 pmol primers, 0.2 mM dNTPs, and 10–50 ng of template DNA in a final volume of 50 μL. The cycle parameters used a low cycle number to reduce chimera production[51], and were as follows: Initial denaturation 95°C 2 min 30 s, then 18 cycles of 95°C 10 s; 55°C 10 s; 72°C 30 s; with final extension of 72°C 2 min. Triplicate PCR reactions were pooled, before size selection by gel extraction with the QIAquick gel extraction kit, concentrated by using MinElute PCR Purification Kit (Qiagen), quantified using Qubit dsDNA HS Assay (Life technologies) before pooling of MID tagged products in equimolar amounts in preparation for multiplex barcode pyrosequencing. Roche 454 GS-FLX sequencing was undertaken at the Centre for Genome Research, University of Liverpool. yielding a total of 3,036,198 reads. Sequences were processed with flows trimmed to 400 and processed/cleaned using PyroDist for distance calculation, FCluster for clustering analysis, PyroNoise in mothur based on AmpliconNoiseV1.25 [23,24] to denoise flowgrams, incorporating Perseus for chimera removal. Sequences were processed with flows trimmed to 400 and processed/cleaned using PyroDist for distance calculation, FCluster for clustering analysis, PyroNoise in mothur based on AmpliconNoiseV1.25 [23,24] Sequences have been deposited at European Nucleotide Archive, accession number PRJEB7724. Sequences were then clustered into OTUs at the 97% similarity level and taxonomy identified using a local 16S rRNA database (Greengenes database release Feb-2011; http://greengenes.lbl.gov) using QIIME's de novo OTU picking scripts [26]. The otus.biom file was ratified to lowest sequence sample size of 9,567 sequences before NMDS statistical analysis was undertaken in R statistical package.

### Antibody Analysis

Analysis of serum parasite-specific IgG2a/c production confirming chronic infection was carried out by capture ELISA. In brief, Immulon IV plates (Dynatech) were coated with T. muris ES Ag (5μg/ml) in carbonate/bicarbonate buffer, pH 9.6, overnight at 4°C. After blocking (3% BSA in PBS, 0.05% Tween), eight serial 2-fold dilutions of sera (from an initial 20-fold dilution) were added to the plates. Serum parasite-specific antibodys were detected using biotinylated rat anti–mouse IgG2a (PharMingen).

### Flow cytometry

Caecum and proximal colon were excised and intra epithelial lymphocytes (IEL) and lamina propria lymphocytes (LILP) were prepared essentially as described [52] with slight ±modification in the tissue digestion step (digestion medium used was RPMI with 10% Foetal calf serum, 0.1% w/v collagenase type I and Dispase II (both Invitrogen), and tissue was digested for 30min at 37°C). Cell suspensions were blocked with anti-FcγR antibody (clone 24G2; eBioscience) and processed with ebioscience fix/perm buffer as per manufacturer’s instructions before labelling with antibodies specific for CD4 (clone GK1.5; eBioscience), Foxp3 (clone FJK-16s; eBioscience) and T-bet (clone TWAJ; eBioscience). All samples were analysed on a FACS LSRII.

### Metabolomics

Weighed subsamples were extracted in 1.2 ml 50:50 MeOH:H2O using a bead homogeniser (Qiagen Tissuelyser) for 10 min at 25 Hz. Samples were then centrifuged 13,000 x g for 15 min, and normalised supernatant volumes (corrected to equivalent sample weight) added to 50 μl Int Std and 600 μl MeOH. Two replicates were prepared per sample, for GC and LC analysis. Diluted supernatant was centrifuged to remove suspended particles and 850 ul of the supernatant was taken and dried overnight in a vacuum concentrator, without heating. 20 pooled QC residues built from 10 subsamples taken through above procedure, the final supernatants were combined, mixed and re-aliquotted prior to drying as above.

### GC-MS analysis

Methoxime/Trimethylsilyl derivatives for GCMS were prepared using a previously described procedure [53] GC-MS analysis was carried out using a Gerstel MPS2 autosampler, an Agilent 7890A Gas Chromatograph with Split/Splitless inlet, and a LECO Pegasus HT time-of-flight mass spectrometer. The method used was based upon that used previously for untargeted metabolomics [49]

### LC-MS analysis

For LC-MS analysis, dried extracts were reconstituted in 120 μl methanol and centrifuged for 15 mins at 13,000 x g before being transferred to autosampler vials. Samples were analysed on an Accela UHPLC system coupled to an Orbitrap Velos mass spectrometer equipped with a heated electrospray ionisation source (HESI) (ThermoFisher Scientific, Hemel Hempstead, UK). Samples were analysed separately in positive- and negative-ion modes. Chromatographic separations were performed on a Hypersil GOLD column (100 x 2.1 mm, 1.9 μm; ThermoFisher Scientific, Runcorn, UK) operating at a column temperature of 50°C. Two solvents were applied (solvent A—0.1% formic acid in water (vol/vol) and solvent B—0.1% formic acid in methanol (vol/vol)) at a flow rate of 400 μL/min. Solvent A was held at 100% for 0.5 minutes followed by an increase to 100% solvent B over 4.5 minutes, which was then held at 100% solvent B for a further 15.5 minutes. A step change to 100% solvent A was performed at 20.5 minutes and then held at 100% solvent A to equilibrate for 1.5 minutes. All column eluent was transferred to the mass spectrometer and full-scan profiling data were acquired in the Orbitrap mass analyser (mass resolution 30,000 at m/z = 400). The source and ion transfer parameters applied were as follows; source heater = 200°C, sheath gas = 50 (arbitrary units), aux gas = 15 (arbitrary units), capillary temperature = 300°C, ISpray voltage = 4.5kV (positive-ion mode) and 3kV (negative-ion mode), slens = 60% (positive-ion mode) 65% (negative-ion mode) and AGC = 5 x 10^5^. XCMS software was employed to convert (or deconvolve) each 3-D data matrix (intensity × m/z × time—one per sample) into a matrix of detected peaks vs sample identification (ID) with peak response for detected metabolites reported, where a peak response is defined as the sum of intensities over a window of specified mass and time ranges. Default settings were employed in XCMS with the exception of S/N threshold (3), step (0.02), m/z diff (0.05) and for grouping bandwidth (10), mzwidth (0.05) and minfrac (0.15). Putative metabolite identification was performed applying the PUTMEDID-LCMS workflows as previously described [54] with a RT window of 3 seconds and a m/z error of 5 ppm.

### Statistics and graphing

Statistics were undertaken using the R-package, with Multivariate analysis was undertaken on these data using the Vegan[21] and Ecodist[55] packages in R. A Non parametric version of Multidimensional scaling (NMDS) was used to assess communities using bray-curtis dissimilarities to characterise the difference between communities. The NMDS figures are plotted in arbitrary two dimensional space with axis indicating Euclidian distance between samples centred on zero, Stress indicates the quality of fit of Bray Curtis dissimilarities, onto two dimensional Euclidian plots (>0.2 is a good fit). Graphing was undertaken using sigma plot (Systat Software Inc).

### Ethics Statement

The program of work was approved by the University of Manchester Animal Welfare and Ethical Review Body and carried out under the regulations and guidelines of the Home Office, Animals (Scientific Procedures Act), 1986, License PPL/70/8127.

## Supporting Information

S1 Fig Caecal Microbial communities assessed by DGGE after *T*.*muris* infection. NMDS analysis of the caecum assessed by 16S rRNA gene DGGE as a result of *T*.*muris* infection. Each point represents an individual mouse, and the *infected individual, post experiment was discovered to have not been infected (no parasites; no parasite specific antibody response), although housed in infected cage demonstrating the robustness of microbial community selection due to treatment. Axis represent scale for Euclidian distance between samples centred on zero, Stress indicates the quality of fit of data (>0.2 is a good fit).
(TIF)

S2 Fig DGGE analysis of the progression of microbial communities on *T*. *muris* infection. NMDS analysis of Microbial communities in stool monitored over time by 16S rRNA gene DGGE as a result of *T*. *muris* infection. Samples were taken as day 0, 14, 28, and 41. NA = naïve, IF = infected. Axis represent scale for Euclidian distance between samples centred on zero, Stress indicates the quality of fit of data (>0.2 is a good fit).
(TIF)

S3 Fig ELISA data confirming IgG2a/c response of infected mice. *T*. *muris* specific serum IgG2a/c levels in infected and naïve mice prior to treatment with mebendazole. Serum taken on day 35. NA1-5 = Naïve mouse control 1–5, IH = Infected mice to be cleared with mebendazole.
(TIF)

S4 Fig Stability of naïve mice microbial communities over time. NMDS plot of changes in DGGE profiles of stool samples in naïve mice over Time, (d41 to d91) demonstrating no time dependant effects (adonis: F_1,23_ = 1.87, p = 0.06). Axis represent scale for Euclidian distance between samples centred on zero, Stress indicates the quality of fit of data (>0.2 is a good fit).
(TIF)

S5 Fig Rarefaction curves demonstrating the sequencing depth of samples. Indicating the number of OTUs at 97% sequence similarity level detected in each treatment and timepoint. Dark blue = Naïve, light blue = Naïve antihelmintic treated, orange = Infected antihelmintic treated, red = Infected, Circle = D0, Triangle = D28, Inverted triangle = D41, Square = D91.
(TIF)

S6 Fig Correlations in bacterial family proportions on NMDS plots. To identify if separation on NMDS plots correlated with proportion of bacterial families, Vectors were plotted onto the NMDS in Fig 6. The direction of the arrows indicate greatest gradient of change. Due to multiple vector plotting, p values of correlations were adjusted by FDR, and corrected regressions were plotted naïve, naïve antihelmintic treated, infected antihelmintic treated and infected, in order of dark blue, light blue, orange, red. Axis represent scale for Euclidian distance between samples centred on zero, Stress indicates the quality of fit of data (>0.2 is a good fit).
(TIF)

S7 Fig Proportions of bacteria at the family level. Average values for each treatment were plotted with only bacterial families that have > 0.1% at one or more treatment/timepoint are labelled. Bacteria that family are not known, but order is, are labelled ** with order.
(TIF)

S8 Fig NMDS analysis of Metabolic profiling of stool samples. Infected and naive mice were compared using a) GC-MS, b) LC-MS positive, c) LC-MS negative Axis represent scale for Euclidian distance between samples centred on zero, Stress indicates the quality of fit of data (>0.2 is a good fit).
(TIF)

S9 Fig Analysis of LILP CD4± T-cells of naive, chronic and Mebendazole treated mice infected with a low dose of *T*. *muris*. (A) LILP Cellularity determined via live cell counts on a haemocytometer with the addition of trypan blue. (B) Percentage of CD4± T-cells within LILP cell population and Foxp3± (C) and T-bet± (D) percentage populations including representative flow cytometry plots. Data (n = 5–10 mice per group) are from two independent experiments performed. *, P<0.05; ***, P<0.005 via ANOVA with Dunnet’s post-test, error bars represent SE of means
(TIF)

S10 Fig Analysis of IEL CD4± T-cells of naive, chronic and Mebendazole treated mice infected with a low dose of *T*. *muris*. (A) IEL Cellularity determined via live cell counts on a haemocytometer with the addition of trypan blue. Percent of CD4± T-cells within IEL cell population positive for Foxp3 (B) and T-bet (C) including representative flow cytometry plots.Data (n = 3–10 mice per group) are from two independent experiments performed. *, P<0.05; ***, P<0.005 via ANOVA with Dunnet’s post-test, error bars represent SE of means.
(TIF)

S1 Table Statistics on NMDS plots of DGGE assessment of communities' profiles. Adonis calculated significant differences on NMDS distance matrices in pairwise comparison between treatments at individual time points. NA = naïve, NH naïve anti helminth treated, IH = infected anti helminth treated, IF = Infected. Bonferroni-adjusted significance level of 0.00833 was calculated and significant values are marked with a *.
(XLSX)

S2 Table Significance values of bacterial Genus that differ as a result of treatment at D91. (XLSX)

S3 Table Metabolomic differences identified between naïve and infected mice at day 41 post infection. (XLS)
