# Supplementary figures and images for: Chronic Trichuris muris Infection in C57BL/6 Mice Causes Significant Changes in Host Microbiota and Metabolome: Effects Reversed by Pathogen Clearance
Source: PLoS One. 2015 May 4;10(5):e0125945. doi: 10.1371/journal.pone.0125945 (PMC4418675; doi:10.1371/journal.pone.0125945)

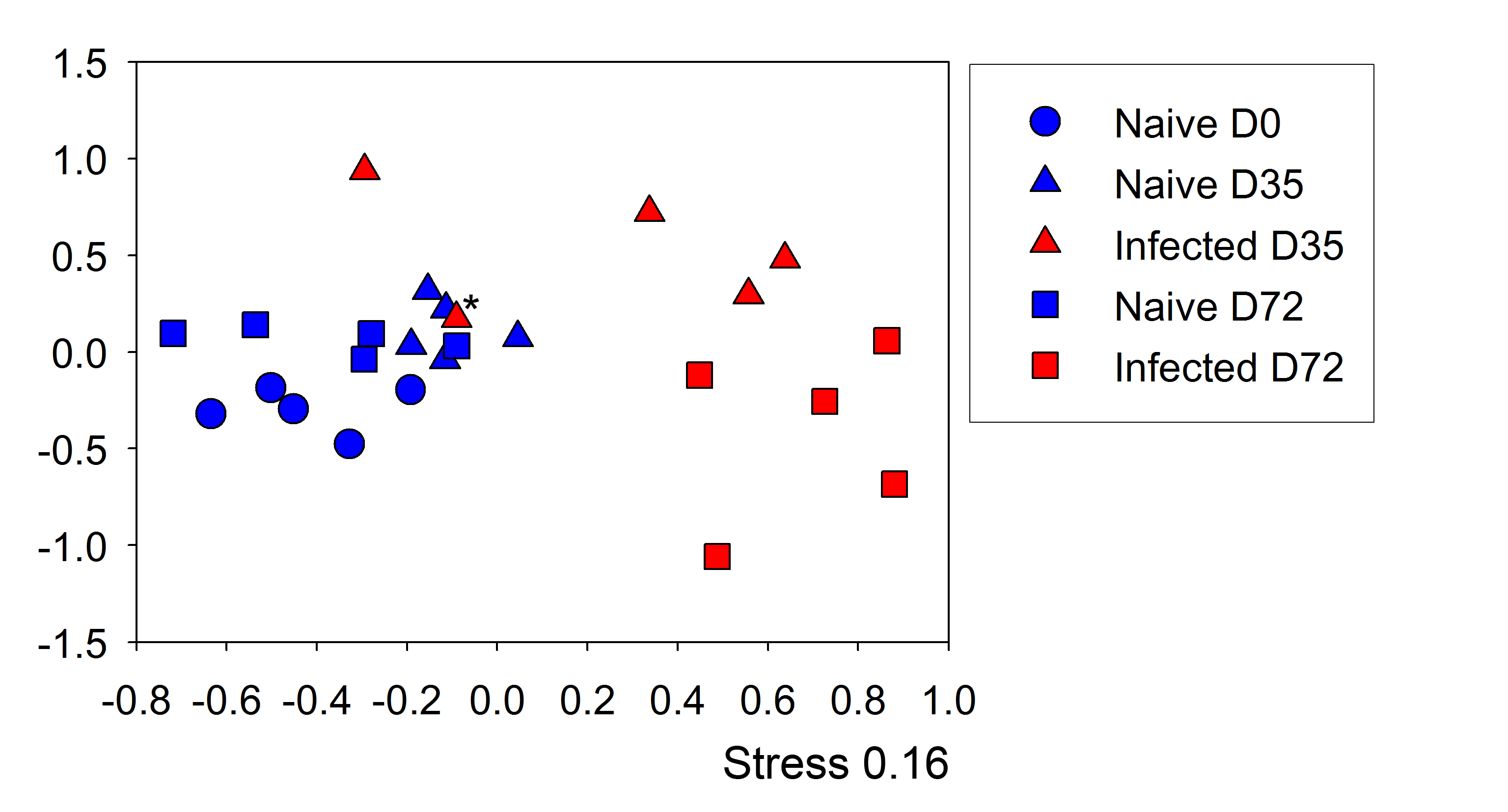

Supplement: S1 Fig — NMDS analysis of the caecum assessed by 16S rRNA gene DGGE as a result of T.muris infection. Each point represents an individual mouse, and the *infected individual, post experiment was discovered to have not been infected (no parasites; no parasite specific antibody response), although housed in infected cage demonstrating the robustness of microbial community selection due to treatment. Axis represent scale for Euclidian distance between samples centred on zero, Stress indicates the quality of fit of data (>0.2 is a good fit). (TIF) [file pone.0125945.s001.tif]

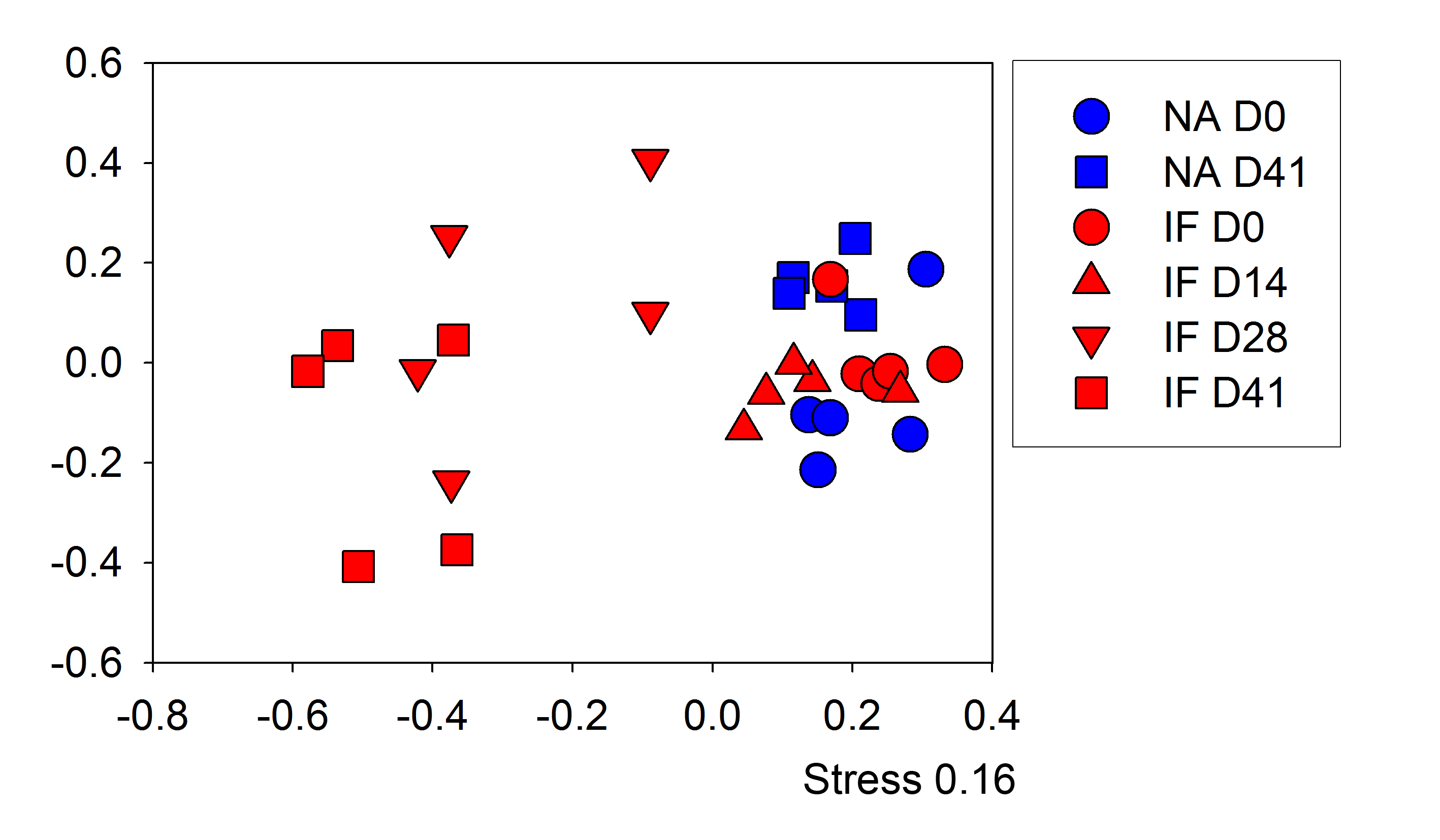

Supplement: S2 Fig — NMDS analysis of Microbial communities in stool monitored over time by 16S rRNA gene DGGE as a result of T. muris infection. Samples were taken as day 0, 14, 28, and 41. NA = naïve, IF = infected. Axis represent scale for Euclidian distance between samples centred on zero, Stress indicates the quality of fit of data (>0.2 is a good fit). (TIF) [file pone.0125945.s002.tif]

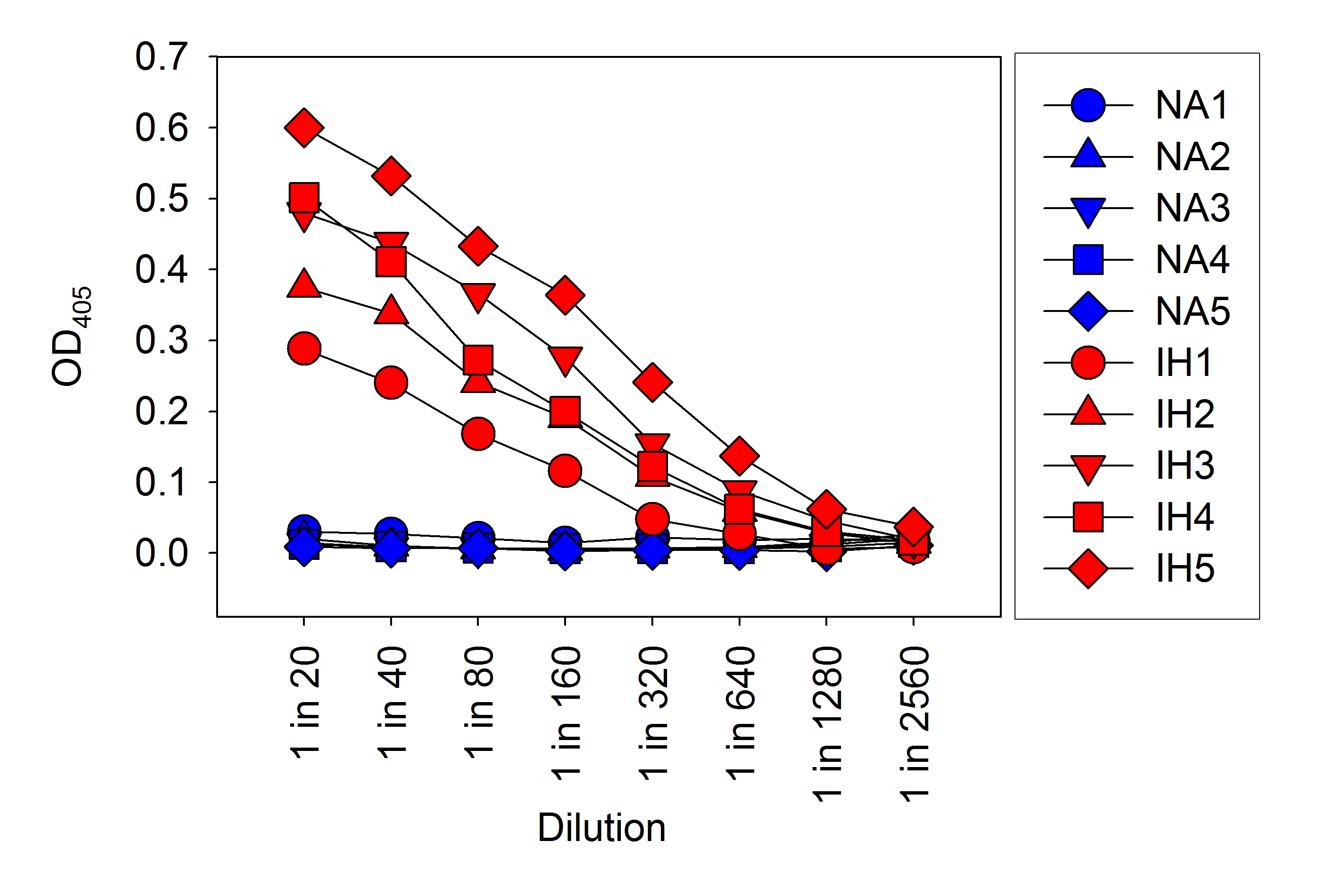

Supplement: S3 Fig — T. muris specific serum IgG2a/c levels in infected and naïve mice prior to treatment with mebendazole. Serum taken on day 35. NA1-5 = Naïve mouse control 1–5, IH = Infected mice to be cleared with mebendazole. (TIF) [file pone.0125945.s003.tif]

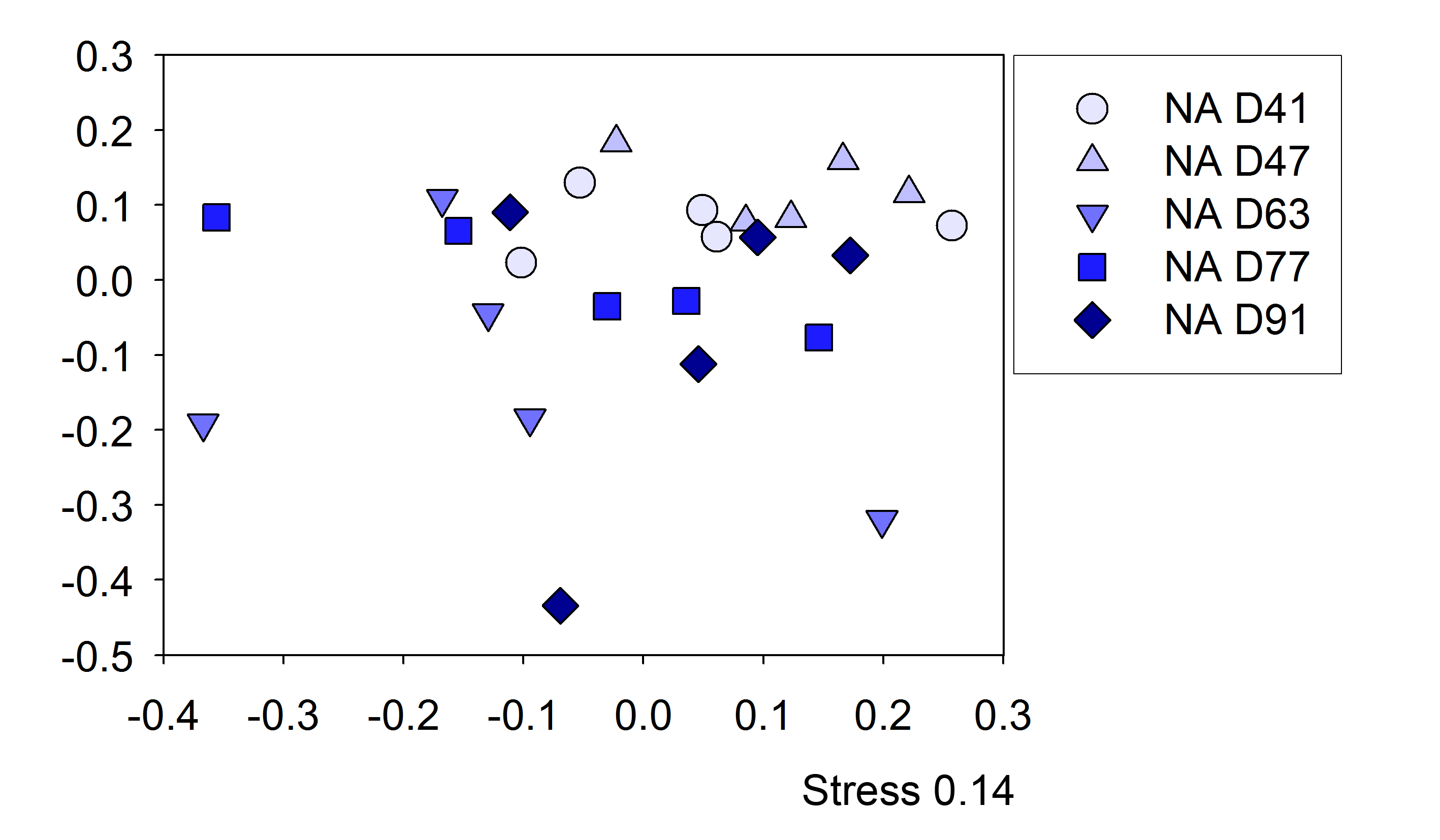

Supplement: S4 Fig — NMDS plot of changes in DGGE profiles of stool samples in naïve mice over Time, (d41 to d91) demonstrating no time dependant effects (adonis: F1,23 = 1.87, p = 0.06). Axis represent scale for Euclidian distance between samples centred on zero, Stress indicates the quality of fit of data (>0.2 is a good fit). (TIF) [file pone.0125945.s004.tif]

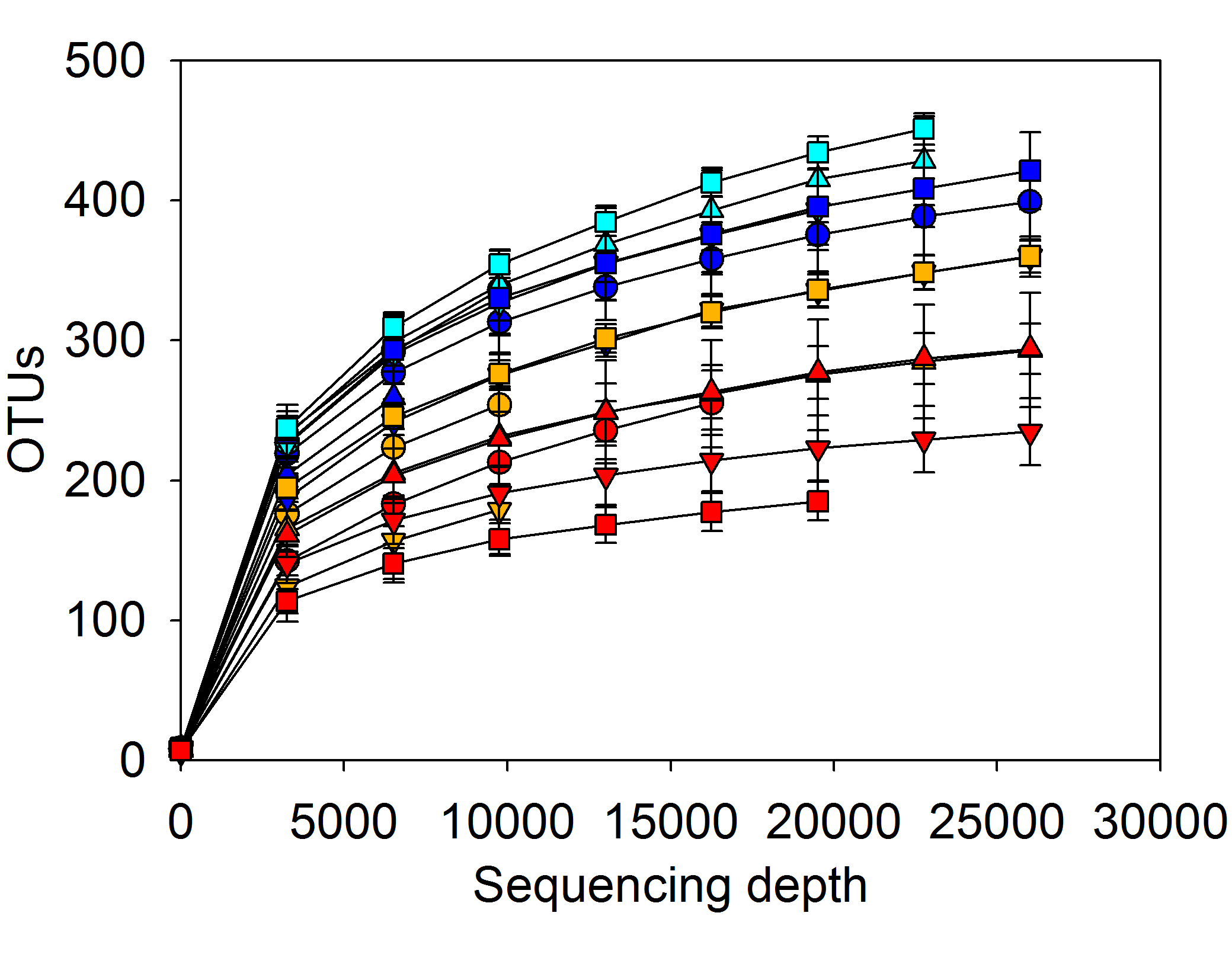

Supplement: S5 Fig — Indicating the number of OTUs at 97% sequence similarity level detected in each treatment and timepoint. Dark blue = Naïve, light blue = Naïve antihelmintic treated, orange = Infected antihelmintic treated, red = Infected, Circle = D0, Triangle = D28, Inverted triangle = D41, Square = D91. (TIF) [file pone.0125945.s005.tif]

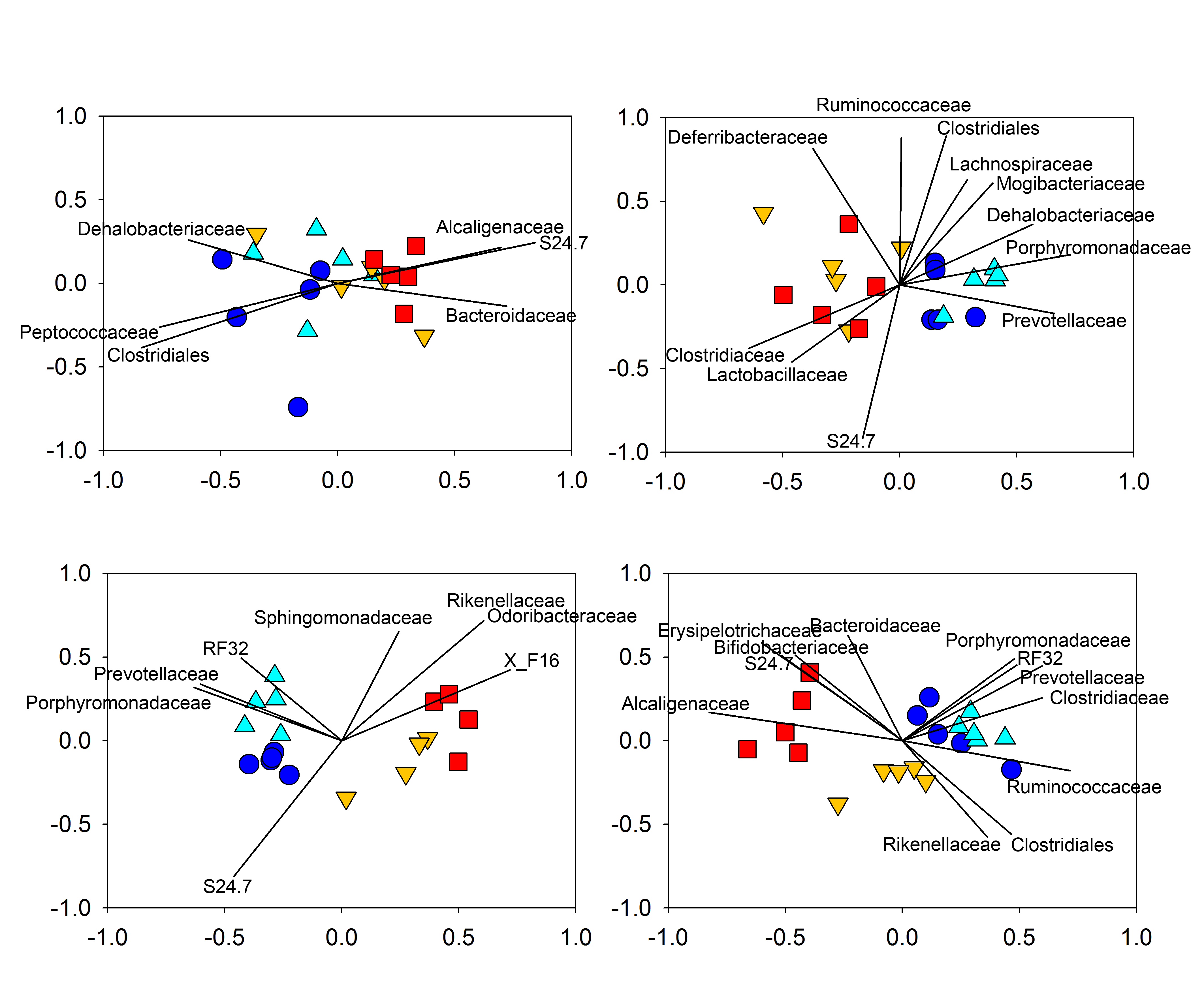

Supplement: S6 Fig — To identify if separation on NMDS plots correlated with proportion of bacterial families, Vectors were plotted onto the NMDS in Fig 6. The direction of the arrows indicate greatest gradient of change. Due to multiple vector plotting, p values of correlations were adjusted by FDR, and corrected regressions were plotted naïve, naïve antihelmintic treated, infected antihelmintic treated and infected, in order of dark blue, light blue, orange, red. Axis represent scale for Euclidian distance between samples centred on zero, Stress indicates the quality of fit of data (>0.2 is a good fit). (TIF) [file pone.0125945.s006.tif]

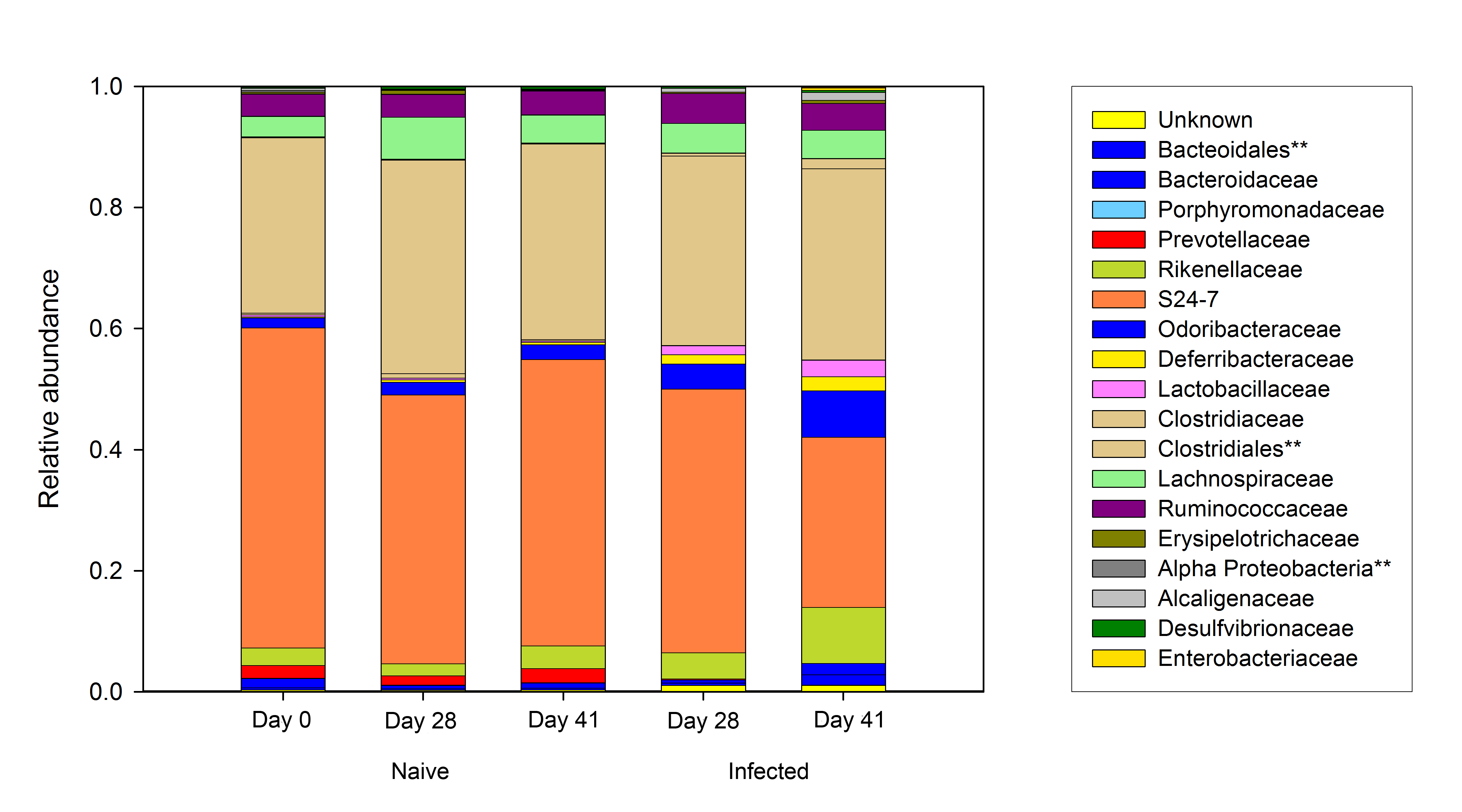

Supplement: S7 Fig — Average values for each treatment were plotted with only bacterial families that have > 0.1% at one or more treatment/timepoint are labelled. Bacteria that family are not known, but order is, are labelled ** with order. (TIF) [file pone.0125945.s007.tif]

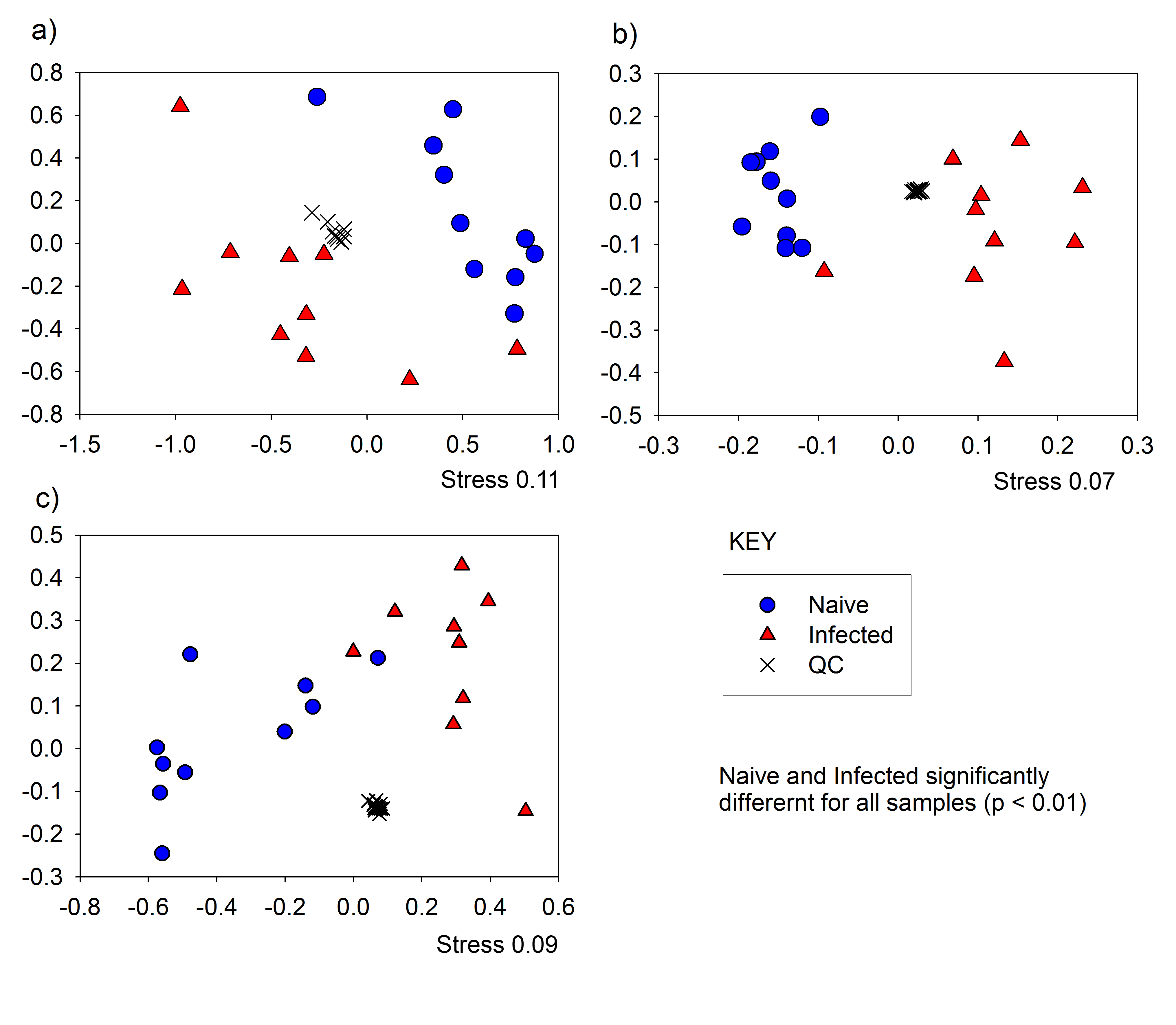

Supplement: S8 Fig — Infected and naive mice were compared using a) GC-MS, b) LC-MS positive, c) LC-MS negative Axis represent scale for Euclidian distance between samples centred on zero, Stress indicates the quality of fit of data (>0.2 is a good fit). (TIF) [file pone.0125945.s008.tif]
